# Chemical structure and bonding in a thorium(iii)–aluminum heterobimetallic complex†
†Electronic supplementary information (ESI) available: Synthetic procedures and characterization information (PDF) and corresponding CIF files (CIF). CCDC 1822996–1822999. For ESI and crystallographic data in CIF or other electronic format see DOI: 10.1039/c8sc01260a


**DOI:** 10.1039/c8sc01260a

**Published:** 2018-04-24

**Authors:** Alison B. Altman, Alexandra C. Brown, Guodong Rao, Trevor D. Lohrey, R. David Britt, Laurent Maron, Stefan G. Minasian, David K. Shuh, John Arnold

**Affiliations:** a Department of Chemistry , University of California , Berkeley , California 94720 , USA; b Chemical Sciences Division , Lawrence Berkeley National Laboratory , Berkeley , California 94720 , USA . Email: arnold@berkeley.edu; c Department of Chemistry , University of California , Davis , California 95616 , USA; d LPCNO , Université de Toulouse , INAS Toulouse , 135 Avenue de Rangueil , 31077 , Toulouse , France

## Abstract

Thorium sits at a unique position on the periodic table. On one hand, there is little evidence that its 5f orbitals engage in bonding as they do in other early actinides; on the other hand, its chemistry is distinct from Lewis acidic transition metals. To gain insight into the underlying electronic structure of Th and develop trends across the actinide series, it is useful to study Th(iii) and Th(ii) systems with valence electrons that may engage in non-electrostatic metal–ligand interactions, although only a handful of such systems are known. To expand the range of low-valent compounds and gain deeper insight into Th electronic structure, we targeted actinide bimetallic complexes containing metal–metal bonds. Herein, we report the syntheses of Th–Al bimetallics from reactions between a di-*tert*-butylcyclopentadienyl supported Th(iv) dihalide (Cp^‡^_2_ThCl_2_) and an anionic aluminum hydride salt [K(H_3_AlC(SiMe_3_)_3_) (**1**)]. Reduction of the [Th(iv)](Cl)–[Al] product resulted in a [Th(iii)]–[Al] complex [Cp^‡^_2_Th(μ-H_3_)AlC(SiMe_3_)_3_ (**4**)]. The U(iii) analogue [Cp^‡^_2_U(μ-H_3_)AlC(SiMe_3_)_3_ (**5**)] could be synthesized directly from a U(iii) halide starting material. Electron paramagnetic resonance studies on **4** demonstrate hyperfine interactions between the unpaired electron and the Al atom indicative of spin density delocalization from the Th metal center to the Al. Density functional theory and atom in molecules calculations confirmed the presence of An→Al interactions in **4** and **5**, which represents the first examples of An→M interactions where the actinide behaves as an electron donor.

## Abstract 2

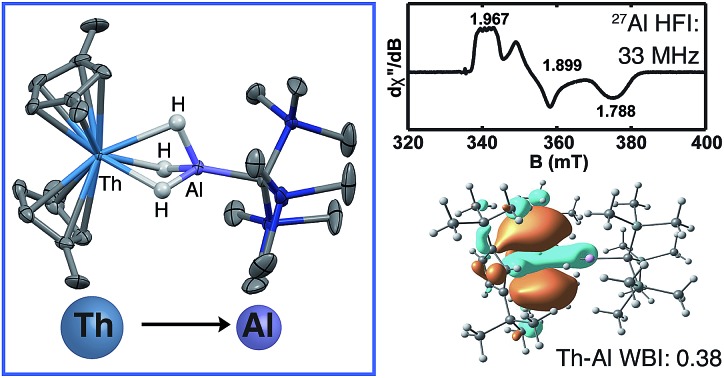
We describe the syntheses of [Th(iii)]–[Al] and [U(iii)]–[Al] bimetallics that demonstrate An→Al interactions where the actinide behaves as an electron donor.

## Introduction

Molecular actinide compounds can house electrons in 5f, 6d, 7p, and 7s orbitals, leading to patterns in chemical reactivity,^1^–^3^ structural motifs^4^–^7^ and magnetic properties^8^,^9^ that are uncommon for metals in other parts of the periodic table. To account for this behaviour, it is necessary to examine how actinide-based electrons are perturbed by ligand field interactions while also considering the effects of strong spin–orbit coupling and significant electron–electron repulsion. However, the interplay between these physical processes is complex and difficult to predict based on models developed for analogous systems comprised of transition metals and lanthanides.^10^–^12^ While in-depth characterization and analysis of known actinides compounds is a productive route for deconvoluting these factors, synthesis of new molecules that diversify the known examples of actinide behaviour is also vital for improving fundamental understanding of actinide chemistry.

Recently, significant progress has been made in exploring the chemistry of thorium complexes containing new bonding motifs and demonstrating novel reactivity.^13^–^20^ In these Th coordination compounds, the metal is well regarded as a Lewis acidic, redox-inactive +4 ion with a large ionic radius and no metal-based electrons, leading to reactivity driven by electrostatic interactions.^21^ Similarly, in solid state complexes, Th 5f orbitals are relatively high in energy so that in materials such as ThO_2_, the bonding is predominantly electrostatic in nature and ThO_2_ is a regular charge-transfer insulator.^22^ Although charge transfer from ligands to Th 5f or 6d orbitals has been observed in some molecular systems, the lack of reactivity driven by Th-based electrons has led to questions over whether thorium's placement within the actinide series is appropriate.^23^–^28^ While atomic thorium exhibits a 6d^2^7s^2^ ground state that mirrors group IV elements, its chemistry is distinct from transition metal analogues.^29^–^31^ These examples highlight how the study of thorium complexes may help to elucidate the subtle but important differences between actinide and transition metal chemistry. However, in complexes where electrostatic interactions dominate Th–L bonding, it remains challenging to distinguish the relevant electronic effects that govern Th chemistry.

In contrast, our understanding of Th chemistry has increased greatly upon discoveries of organometallics with thorium formally in the +3 and +2 oxidation state.^32^ Th(iii) and Th(ii) complexes are potentially good candidates for providing insight into Th reactivity and performing new redox transformations in analogy to the recent progress associated with the study of low-valent U complexes.^33^–^35^ However, the large energetic requirements for the reduction from Th(iv) materials (calculated for ThCl_6_^2–^ to be +3.7 V *vs.* NHE relative to +0.6 V for U(iv)/(iii) reduction in UCl_6_^2–^) inhibits the study of Th(iii) and Th(ii) complexes.^36^ To date, there are only nine structurally characterized examples of low-valent Th containing molecules reported in the literature.^37^–^44^ While these systems all exhibit 6d^1^ or 6d^2^ ground states, the symmetry of the coordination environment is such that the d-electrons are essentially non-bonding, which prevents destabilizing interactions with electron donating ligands.^37^,^39^,^45^–^48^ Synthesizing Th(ii) or Th(iii) complexes with new structural motifs may promote 5f or 6d orbital involvement in bonding, expanding upon the known chemistry of thorium and providing new insight into actinide electronic structure.

Because formation of metal–metal bonds is known to stabilize low-valent metal systems in general,^49^ we pursued synthetic routes to new Th(iii) compounds containing Th–M interactions. Although all known examples of bimetallics with Th–M bonds rely on M→Th(iv) donor–acceptor motifs,^50^–^53^ we hypothesized that a Th(iii) complex could be stabilized *via* electron delocalization from thorium to the other metal center, similar to the way that redox active ligands acting as electron reservoirs have been shown to expand the redox chemistry of Th complexes while maintaining some low-valent Th character.^54^ Evans and coworkers reported the first examples of Th(iii) bimetallics containing a Th(iii)–μ-H–Th(iv) moiety; however, evidence for Th–Th interactions was not obtained from analysis of the solid structure or using Density functional theory (DFT) calculations.^43^ Building on other synthetic routes to Th hydride complexes,^55^–^60^ we turned to aluminum hydrides to install a Lewis acidic metal in close proximity to the Th center in order to promote a Th→Al interaction. Analogously, Lu and coworkers have utilized TM→Al (TM = Fe, Co, Ni) interactions to enable new chemistry in electron rich transition metal systems.^61^,^62^ Here we report the synthesis and characterization of Th–Al and U–Al heterobimetallics using an alanate ligand recently found to support a Ti–Al bimetallic.^63^ Electron paramagnetic resonance (EPR) studies of the Th(iii) complex demonstrated significant contributions from Al valence orbitals to the singly occupied molecular orbital (SOMO) compared with the Ti(iii) analogue. A comprehensive DFT investigation confirmed a new metal–metal interaction paradigm that has not been observed previously for actinides, involving electron donation from the 6d-orbitals in the Th(iii)–Al complex and from the 5f orbitals in the U(iii)–Al complex.

## Results and discussion

### Synthesis of Cp^‡^_2_ThCl(μ-H)_3_AlC(SiMe_3_)_3_ (**2**) and Cp^‡^_2_Th(H)(μ-H)_3_AlC(SiMe_3_)_3_ (**3**)

A [Th(iv)](Cl)–[Al] starting material was targeted as a precursor to Th(iii) bimetallics. Salt-metathesis pathways were employed using the alanate ligand K[H_3_AlC(SiMe_3_)_3_] (**1**) and Th dihalides. Reactions of **1** with Cp^‡^_2_ThCl_2_ (Cp^‡^ = di-*tert*-butylcyclopentadienyl) resulted in a mixture of two products: Cp^‡^_2_ThCl(μ-H)_3_AlC(SiMe_3_)_3_ (**2**), and Cp^‡^_2_Th(H)(μ-H)_3_AlC(SiMe_3_)_3_ (**3**). The ^1^H NMR spectra of **2** and **3** were similar but distinct implying analogous solution state structures with more deshielded resonances in **2** relative to **3** resulting from the greater electronegativity of the chloride compared with the hydride ligand. The ^1^H NMR spectrum of **3** also contained an additional resonance at 15.11 ppm attributed to the terminal Th–*H* hydride, which is in the range reported for other known examples.^43^,^56^,^64^–^67^

**Scheme 1:**
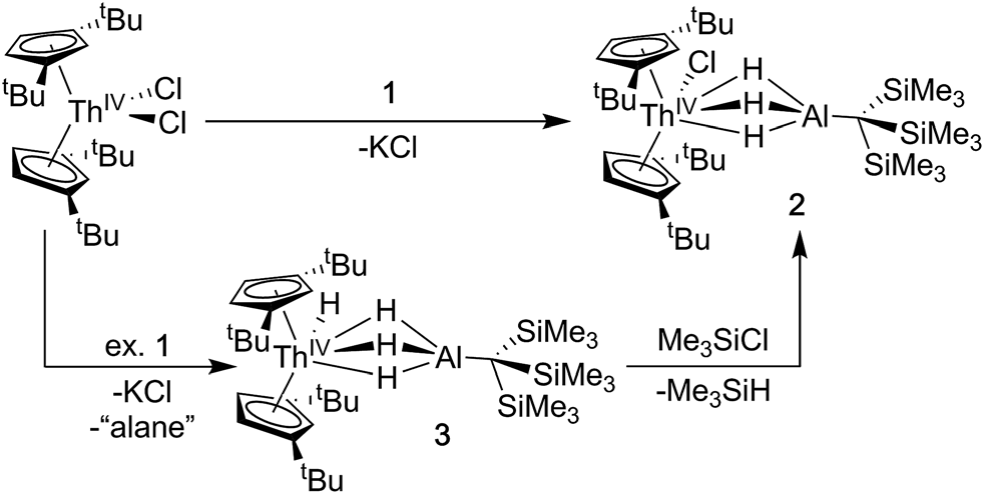
Synthesis of **2** and **3**.

Further ^1^H NMR experiments demonstrated that **2** could be converted to **3** upon addition of excess **1**, suggesting the alanate ligand could also access hydride transfer pathways, although the expected alane side product was not observed in NMR-scale reactions. Conversely, **3** reacted with chlorotrimethylsilane to form **2** and trimethylsilane. This reactivity could be harnessed to generate analytically pure **2** in good yield by stirring crude mixtures of **2** and **3** with a commensurate amount of chlorotrimethylsilane as determined by ^1^H NMR spectroscopy, followed by removal of the volatile silane yielding a white solid (Scheme 1). Compound **3** could also be isolated in moderate yield by refluxing mixtures of Cp^‡^_2_ThCl_2_ and excess **1** followed by extraction of the crude product with *n*-hexane.

**Fig. 1:**
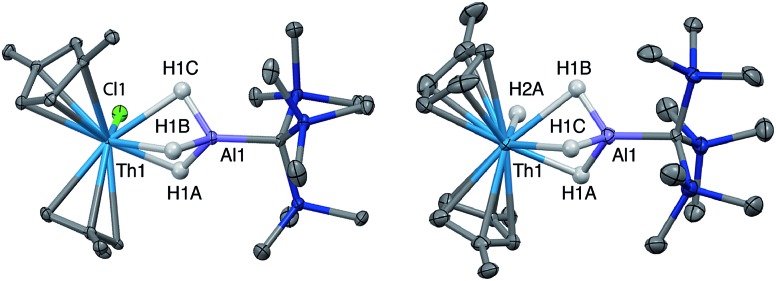
Crystallographically determined structures of **2** (left) and **3** (right). Thermal ellipsoids are shown at the 50% probability level. C–H hydrogens and *tert*-butyl methyl groups were omitted for clarity. Metal hydrides were located in the Fourier difference map and their positions were freely refined.

Crystalline samples of **2** and **3** could be isolated from concentrated hexamethyldisiloxane (HMDSO), and their solid-state structures were determined with single crystal X-ray diffraction studies (Fig. 1). The coordination and geometry of both complexes was found to be nearly identical, with the slight differences in bonding parameters being attributable to the smaller size of the hydride ligand in **3** as compared to the chloride ligand in **2**. This increase in sterics in **2***versus***3** is perhaps best demonstrated by the difference in the Cp^‡^_centroid_–Th–Cp^‡^_centroid_ angles, which were measured to be 117.34(5)° and 121.97(11)°, respectively (Table 1). The increased bending of the metallocene fragment in **2** is a direct consequence of the greater steric encumbrance around the Th center as compared to that in **3**. The crystal structure of the Th terminal hydride complex **3** is of note as there are few crystallographically characterized examples of complexes containing this moiety.^43^,^64^–^68^ Most importantly, the solid-state structures of **2** and **3** provide confirmation as to the binding mode of the H_3_AlC(SiMe_3_)_3_ ligand, which was found in both structures to bind to the Th center through all three hydrides of the alanate. All bridging and terminal hydride atoms in the structures of **2** and **3** were explicitly located and refined freely.

**Table 1:** Selected metrical data from the solid-state structures of actinide–aluminum bimetallics

Bond distance/angle	**2**	**3**	**4**	**5**
Cp^‡^_cent_–M (Å)	2.5534(15), 2.5356(14)	2.530(4), 2.522(4)	2.516(3), 2.477(2)	2.4836(11), 2.4951(11)
Cp^‡^_cent_–M–Cp^‡^_cent_ (°)	117.34(5)	121.97(11)	121.08(8)	121.31(3)
M–Al (Å)	2.976(1)	2.963(3)	2.942(2)	2.940(1)

### Synthesis of Cp^‡^_2_Th(μ-H)_3_AlC(SiMe_3_)_3_ (**4**)

**Scheme 2:**
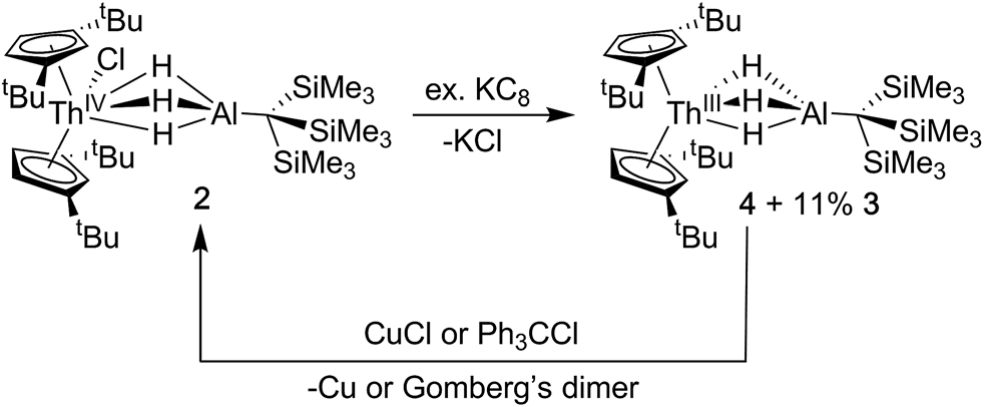
Synthesis and reactivity of **4**.

The addition of KC_8_ to solutions of **2** in toluene, diethyl ether or *n*-hexane produced a rapid color change from colorless to dark purple, and purple crystals were isolated following extraction and crystallization from HMDSO (Scheme 2). The ^1^H NMR spectrum revealed a paramagnetic complex, which was initially formulated as a new Th(iii) bimetallic complex, Cp^‡^_2_Th(μ-H)_3_AlC(SiMe_3_)_3_ (**4**). Despite numerous efforts, the ^1^H NMR spectrum of samples of **4** also demonstrated the persistent presence of small amounts of Cp^‡^_2_Th(H)(μ-H)_3_AlC(SiMe_3_)_3_ (**3**) as a reaction side product. The generation of Th(iv) hydrides is known in the reduction chemistry of Th(iv) to Th(iii) complexes, although the pathways for their formation remain poorly understood.^44^ We found the ratio of **4** : **3** did not change upon recrystallization or across independent syntheses under the same conditions but did vary depending on whether toluene, diethyl ether, or hexane was used during the reduction, with hexane giving optimal results (crystalline material isolated containing at most ∼10% of **3**). Based on integration of the ^1^H NMR resonances, the observed ratio of **4** : **3** also did not vary with changes in temperature from –60 to 100 °C. The ^1^H NMR resonances assigned to **4** also shifted according to the Curie law, implying that there were no significant temperature dependent equilibria over this temperature range that would require further consideration.^69^

**Fig. 2:**
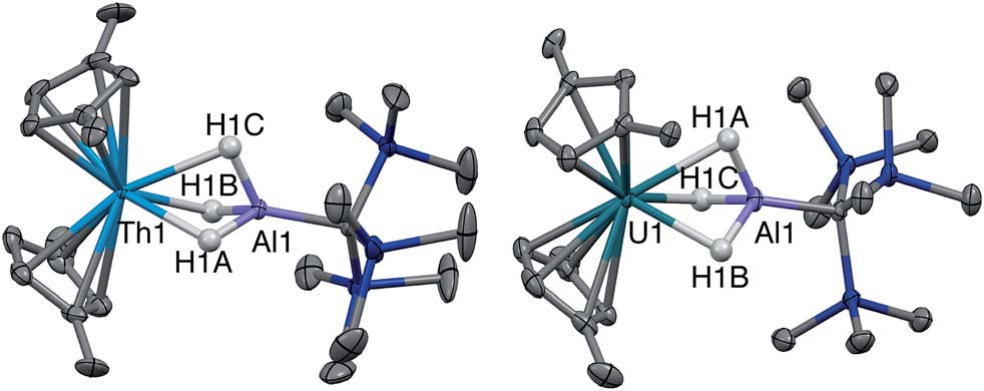
Crystallographically determined structures of **4** (left) and **5** (right). Thermal ellipsoids are shown at the 50% probability level. C–H hydrogens and *tert*-butyl methyl groups were omitted for clarity. Metal hydrides were located in the Fourier difference map and their positions were freely refined.

Single crystal X-ray diffraction studies confirmed the solid-state structure of **4** was similar to **3** overall, although all bond distances decreased by 0.01–0.03 Å in **4** (Fig. 2). While the opposite effect is normally expected upon a reduction of a metal center, this trend was attributed to the steric influence of the hydride ligand. As stated, the ratio of **4** : **3** in samples of isolated **4** did not change upon recrystallization, indicating that the two complexes likely cocrystallized, and that some amount of **3** was present in the single crystal of **4** selected for X-ray diffraction studies. While quantifying the exact amount of **3** present in the single crystal of **4** was not possible based on the diffraction data alone, we believe the structural model of **4** remains valid. In addition to the fact that **3** and **4** were found to crystallize in different space groups of different centering, this stance is supported by the observation that the changes in metrics between the structures of **3** and **4** are on the same order as was measured for the other four examples of crystallographically characterized isostructural Th(iii)/Th(iv)–H pairs Cp^‡^_3_Th/Cp^‡^_3_ThH,^65^,^70^ Cp*_3_Th/Cp*_3_ThH,^44^,^66^ Cp′′_3_Th/Cp′′_3_ThH,^43^,^71^ and Cp^4Me^_3_Th/Cp^4Me^_3_ThH^41^ (where pentamethylcyclopentadienyl = Cp*, bis(trimethylsilyl)cyclopentadienyl = Cp′′, and tetramethylcyclopentadienyl = Cp^4Me^). In all, the solid-state structures of **3** and **4** continue to suggest that Th–ligand bond distances are not sensitive to thorium oxidation state.

Because the structural parameters did not vary significantly across this series and because **4** was consistently isolated as a mixture, we sought additional characterization that confirmed its formulation as a Th(iii) complex. The magnetic moment of **4** was determined by the Evans NMR method to be 1.83 *μ*_B_ at room temperature after applying a diamagnetic correction (*cf.* the calculated spin-only value = 1.73 *μ*_B_). Additionally, the UV-vis absorption spectrum for **4** was similar in line shape and intensity to that observed for Cp^‡^_3_Th and Cp′′_3_Th which both demonstrated three intense features between 450–680 nm with a maximum extinction coefficient (*ε*) of 7700 M^–1^ cm^–1^ (513 nm) and 5100 M^–1^ cm^–1^ (654 nm), in their respective UV-vis spectra.^44^,^72^ The multiple intense features of **4** (360 nm, 1694 M^–1^ cm^–1^; 520 nm, 3999 M^–1^ cm^–1^; 585 nm, 2150 M^–1^ cm^–1^; 640 nm, 2482 M^–1^ cm^–1^) were therefore similarly attributed to dipole allowed 6d → 5f transitions, suggesting a 6d^1^ ground state. The redox chemistry of **4** was also consistent with the proposed radical Th(iii) character (Scheme 2). For example, stoichiometric amounts of copper(i)chloride were found to readily oxidize **4** to **2**. Similarly, addition of chlorotriphenylmethane converted mixtures of **3** and **4** into **2** as well as triphenylmethane and Gomberg's dimer. Taken together with the observation of a paramagnetic species in the NMR and EPR (see below), these observations strongly support our formulation of **4** as a Th(iii) compound.

### Synthesis of Cp^‡^_2_U(μ-H)_3_AlC(SiMe_3_)_3_ (**5**)

**Scheme 3:**
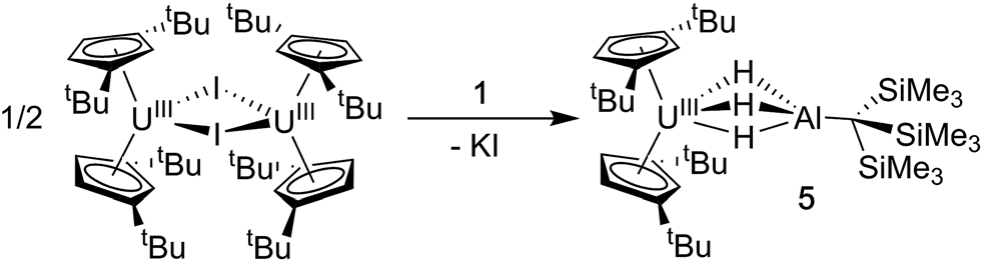
Synthesis of **5**.

We also explored synthetic pathways to a U(iii) analogue of **4** to compare the effects of f-orbital involvement in bonding. Cp^‡^_2_U(μ-H_3_)AlC(SiMe_3_)_3_ (**5**) was readily synthesized by the reaction of trivalent [Cp^‡^_2_UI]_2_ with a slight excess of **1** in toluene (Scheme 3). The resulting dark yellow compound was very soluble in non-coordinating solvents and could be isolated in moderate yield (51%) from HMDSO as crystalline material. A single crystal of **5** was crystallographically characterized and determined to be structurally analogous to **4**. The room temperature magnetic moment, UV-vis and EPR spectra were consistent with the formation of a U(iii) 5f^3^ system (see below).

### EPR spectroscopy

**Fig. 3:**
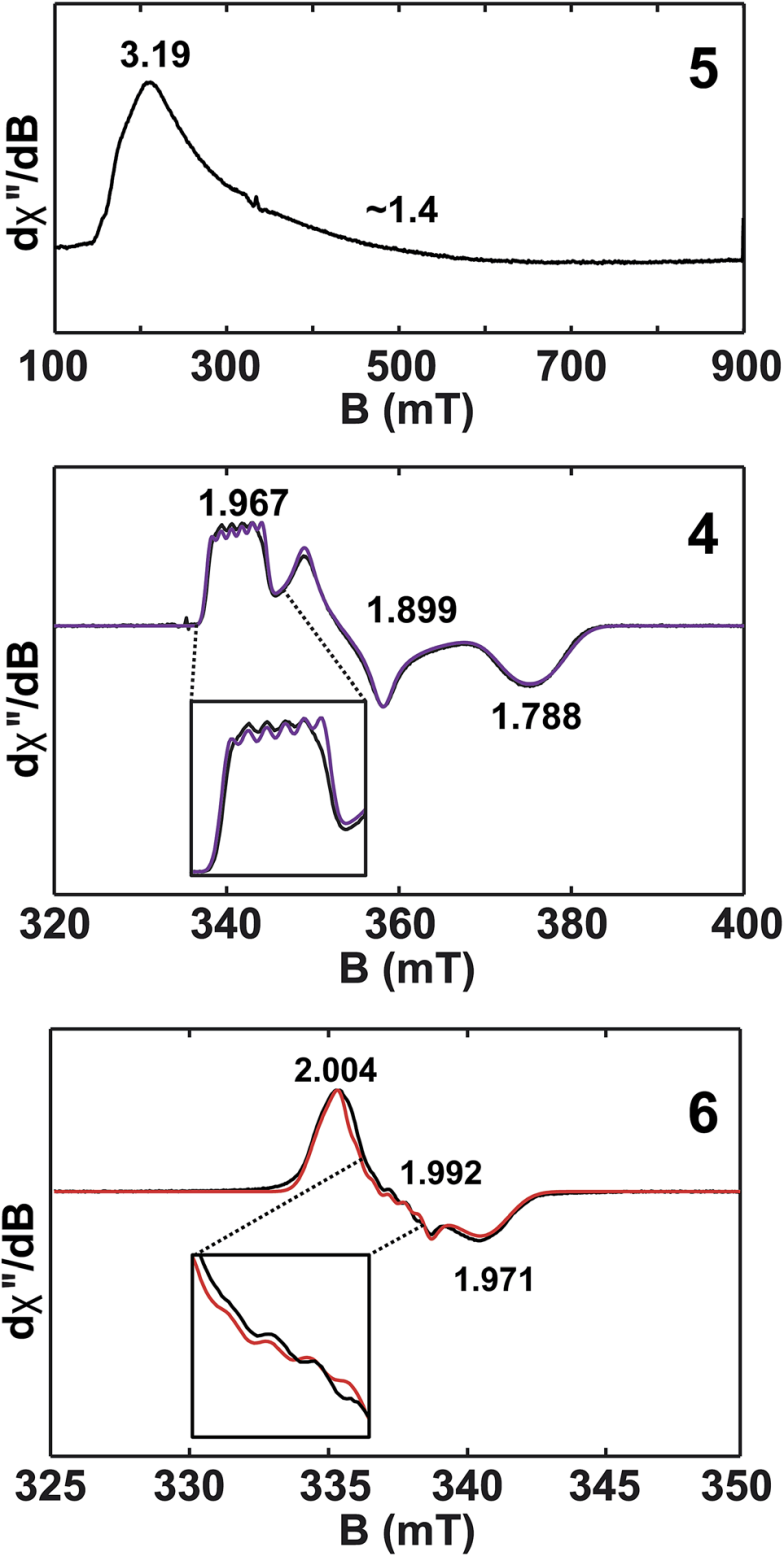
X-band (9.4 GHz) CW-EPR frozen solution spectra of **5** (20 mM in toluene) and **4**, **6** (2 mM in toluene). Observed EPR spectra of **4** and **6** (black traces) overlaid with simulations (purple and red traces, respectively) with **g** = [1.967, 1.899, 1.788] and **A**^27^Al = [33, 40, 33] MHz and **g** = [2.004, 1.992, 1.971] and **A**^27^Al = [8, 16, 8] MHz for **4** and **6**, respectively. Insets show hyperfine splitting. See ESI† for more spectra.

Given the close proximity of the electron rich Th(iii) and U(iii) atoms to the Lewis acidic Al(iii)-based ligands, we hypothesized that **4** and **5** were stabilized by the presence of actinide–aluminum bonds. Although the crystallographically determined M–Al bond distances of these complexes fell within the sum of the covalent radii for the metals [3.01 Å (Th–Al) and 3.17 Å (U–Al)]^73^ they were found to differ only slightly across oxidation state and identity of the actinide [An–Al (Å) = 2.976(1) Å, 2.963(3) Å, 2.942(2) Å, and 2.940(1) Å for **2**, **3**, **4**, and **5**, respectively] suggesting that the interatomic distances were not a reliable metric of actinide–aluminum bonding. To gain insight into whether any actinide–aluminum bond was present in **4** and **5**, we explored spectroscopic and theoretical techniques that probed mixing between the Al 3p and actinide 6d/5f orbitals. Frozen solution EPR spectra for **4** and **5** as well as a reported titanium analogue, Cp_2_Ti(iii)(μ-H)_2_Al(H)C(SiMe_3_)_3_ (**6**),^63^ are presented in Fig. 3. As is generally expected, the EPR signal of **5** was only observed below 20 K due to the fast relaxation of f electrons.^39^ The *g*_1_ ∼ 3.2 was comparable to values observed for other 5f^3^ U(iii) examples with a ^4^I_9/2_ ground term,^42^,^53^ although *g*_2_ was poorly-resolved. In contrast, the EPR signal of **4** and **6** (*T* = 50 K) consisted of well-separated rhombic **g** tensors (**g** = [1.967, 1.899, 1.788] and [2.004, 1.992, 1.971] for **4** and **6**, respectively), which was further confirmed for **4** by Q-band electron spin-echo detected EPR spectrum (Fig. S14†). Both spectra were consistent with a d^1^ configuration and d_*z*_^2^ ground state (*g*_*z*_ ∼ *g*_e_ > *g*_*x*,*y*_), with the greater separation of principal *g* values and deviation from *g*_e_ (2.0023) in complex **4** caused by the much larger spin–orbit coupling for trivalent thorium. Moreover, we observed well-resolved hyperfine splitting at the *g*_1_ region of the EPR spectrum of **4**, with a sextet pattern and a coupling constant ∼33 MHz, which was assigned to ^27^Al (*I* = 5/2) hyperfine interactions (HFI). Broadening in other regions of the spectrum indicated ^27^Al hyperfine splittings of similar magnitude which were reproduced by spectral simulation suggesting an *a*_iso_^27^Al of ∼35 MHz (Fig. 3). This represents one of the few reported ^27^Al HFI in a molecular system.^74^–^76^ Similar splitting was also observed for **6**, albeit with a smaller *a*_iso_ of ∼10 MHz, consistent with values found in another Ti(iii)–Al bimetallic system.^75^ The >3× larger ^27^Al HFI found in **4** thus shows a greater amount of spin-delocalization on the Al atom in **4** compared with **6**. This is attributed to an increased amount of orbital mixing between the Th and Al orbitals as suggested by the electronic structure calculations (see below). Pulsed EPR studies that further quantify bonding in this system will be the focus of future work.

### Electronic structure calculations

**Fig. 4:**
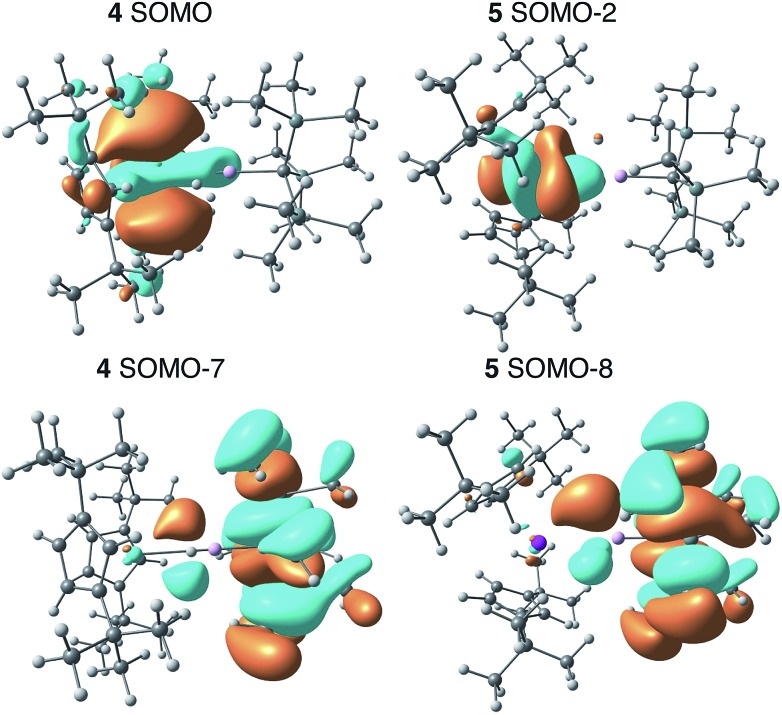
Calculated frontier orbitals demonstrating M–Al bonding character for **4** (left) and **5** (right).

We employed DFT to gain further insight into the nature of the interaction between the Th(iii) and Al(iii) centers of **4**. Visualizing the valence orbitals confirmed that the unpaired electron of **4** and the Ti analogue **6** resided in a singly occupied molecular orbital (SOMO) of d_*z*_^2^ character that was aligned along the axis perpendicular to the pseudo mirror plane. No evidence of 5f-orbital contribution to the SOMO was observed for **4**. In contrast, the three highest occupied SOMOs of **5** were 5f orbitals (Fig. 4 and S17†).

Further examination of the valence orbitals identified a π type bonding interaction between the Al 3p (12% in **4** and 10% in **5**) and An 6d or 5f orbitals in the SOMO–7 and SOMO–8 for **4** (88% 6d) and **5** (90% 5f), respectively; no Al 3p and Ti 3d bonding interaction was observed for **6**. The strength of the second order donor–acceptor interaction in the Natural Bonding Orbital (NBO) analysis (see computation details in the ESI†) was calculated to be 20.1 kcal mol^–1^ for **4** and 15.0 kcal mol^–1^ for **5**, which is consistent with other calculated dative bond strengths in actinide systems.^77^ Furthermore, Wiberg Bond Indices (WBIs) between the actinide and aluminum for **4** and **5** were found to be 0.38 and 0.29 between the actinide and aluminum, respectively. In comparison, a WBI of 0.07 was found for the Ti–Al interactions in **6**. While these values are smaller than would be expected for full covalent bonds (each covalently shared electron corresponds to a Wiberg bond index of 0.5), they are similar in magnitude to previous examples of interactions between electron-rich metals and actinides in which the other metal acted as a Lewis base and the actinide was an electron acceptor. For, example the Cu(i)→Th(iv) bond in Cp*_2_ThI[N(mesityl)Cu(DMAP)] (DMAP = 4-dimethylaminopyridine) had a WBI of 0.30 and the Al(i)→U(iii) bond in Cp′_3_U–AlCp* had a WBI of 0.487.^53^,^78^ The actinide–aluminum interactions in **4** and **5** are perhaps most remarkable in that they reverse the standard donor–acceptor paradigm for metal–metal bonds involving actinides by exhibiting charge transfer away from the actinide towards the Al-based ligand. Second order NBO analysis confirmed donation from the occupied Th 6d (**4**) or U 5f (**5**) orbitals to an Al 3p orbital, that is an antibonding Al–R orbital with significant Al 3p character.

Finally, atoms in molecules (AIM) methods were used to further scrutinize the Th–Al interaction in **4**. A Bond Critical Point (BCP) was located in between the Th and Al indicating a direct interaction between the two atoms. Analysis of the BCP showed that the calculated electron density at the BCP is 0.04 with a positive Laplacian, similar in magnitude to previous reports for other Th–L bonds (L = Se, Se in Th[E_2_PPh_2_]_4_, Cp in Cp_3_Th and PH_2_ in Th[Tren][PH_2_]).^79^–^81^ Additionally, three Ring Critical Points (RCP) were also located in between each Th–H–Al junction. These results indicate that the Th–Al interaction in **4** comes from the superposition of both bridging-hydrogen mediated interactions as well as a direct metal–metal interaction, consistent with the calculated MOs and NBO analyses.

Together, the EPR and calculated results suggest that the amount of M→Al charge transfer increased from **6** < **5** < **4**. While the significant difference between **6** and **4** can be understood in terms of larger orbital overlap for the more diffuse Th 6d orbitals compared with the Ti 3d orbitals; the similarity between the WBIs and bond energies for **4** and **5** is surprising given their different valence orbital type and occupation (6d^1^*vs.* 5f^3^). Furthermore, Formanuik *et al.* recently demonstrated a ∼3-fold increase in covalency between the cyclopentadienyl ligands and the metal from Cp^‡^_3_Th to Cp^‡^_3_U, which was attributed to the better symmetry driven overlap of the 5f *vs.* 6d An orbitals with the three Cp^‡^ ligands.^70^ In comparison, this study demonstrates that the alanate ligand imparts unique electronic effects to this system, perhaps due to the high energy of its valence orbitals. Spectroscopic and theoretical efforts to further understand and quantify the nature of the M–Al interactions in this system will be the focus of future work.

## Conclusions

We have discovered synthetic pathways to low-valent Th–Al and U–Al bimetallics. While structural investigations confirmed the presence of a M–μH–Al motif with small structural differences across metals and oxidations states, EPR studies of the Th(iii) species showed evidence of Th 6d to Al 3p electron donation that was approximately 3 times greater than the Ti 3d to Al 3p donation observed in the titanium analogue. Calculations confirmed a metal–metal interaction in the Th the U complexes. To the best of our knowledge, this represents the first example of an An→M dative interaction. Our future work will explore whether the incorporation of other Lewis acidic motifs may be used to expand the chemistry of low-valent thorium, or actinide metal bonding more generally.

## Conflicts of interest

There are no conflicts to declare. This report was prepared as an account of work sponsored by an agency of the United States Government. Neither the United States Government nor any agency thereof, nor any of their employees, makes any warranty, express or implied, or assumes any legal liability or responsibility for the accuracy, completeness, or usefulness of any information, apparatus, product, or process disclosed, or represents that its use would not infringe privately owned rights. Reference herein to any specific commercial product, process, or service by trade name, trademark, manufacturer, or otherwise does not necessarily constitute or imply its endorsement, recommendation, or favoring by the United States Government or any agency thereof. The views and opinions of authors expressed herein do not necessarily state or reflect those of the United States Government or any agency thereof.

## Supplementary Material

Supplementary information

Crystal structure data
