# Supplementary material for: Chemical structure and bonding in a thorium(iii)–aluminum heterobimetallic complex
Source: Chem Sci. 2018 Apr 24;9(18):4317–24. doi: 10.1039/c8sc01260a (PMC5944380; doi:10.1039/c8sc01260a)
Supplement: Supplementary file 1 [file SC-009-C8SC01260A-s001.pdf]

Electronic Supplementary Material (ESI) for Chemical Science.
This journal is © The Royal Society of Chemistry 2018

Chemical Structure and Bonding in a Thorium(III)-Aluminum Heterobimetallic Complex

Alison B. Altman,[1,2] Alexandra C. Brown,[1] Guodong Rao,[3] Trevor D. Lohrey,[1,2] R. David Britt,[3] Laurent Maron,[4] Stefan G. Minasian[2], David K. Shuh,[2] John Arnold[1,2]

[1] Department of Chemistry, University of California, Berkeley, California, 94720; [2]Chemical Sciences Division, Lawrence Berkeley National Laboratory, Berkeley, California 94720; [3]Department of Chemistry, University of California, Davis, California, 95616; [4]LPCNO, Université de Toulouse, INSA Toulouse, 135 Avenue de Rangueil, 31077 Toulouse France

Supporting Information

A. Synthetic Details S2
B. NMR Spectra S5
C. Infrared Spectra S11
D. UV-Vis Spectra S12
E. Crystallographic Details S13
F. Electron Paramagnetic Resonance Spectroscopy Details S14
G. Computational Details S16
H. References S23

A. Synthetic Details

General Synthetic Details. All reactions were performed under an inert atmosphere either using standard Schlenk technique or in a MBraun glovebox (<0.5 ppm O_2/H_2O). Toluene, *n*-hexane, diethyl ether (Et_2O), tetrahydrofuran (THF) and dimethoxyethane (DME) were dried and degassed using the commercially available Phoenix Solvent Drying System from JC Meyer Solvent Systems. Hexamethyldisiloxane (HMDSO), C_6D_6, and d^8-toluene were dried over sodium/benzophenone and either vacuum transferred to a storage flask or distilled under N_2. Solution NMR spectra were collected on either Bruker AV-500, AV-600 or DRX-500 spectrometers. ^1H and $^{13}C\{^1H\}$ NMR chemical shifts (δ in ppm) were calibrated to residual solvent peaks. ^{27}Al NMR chemical shifts were referenced to an external standard of 1 M $Al(NO_3)_3$ in H_2O/D_2O (δ= 0 ppm). FT-IR samples were prepared as Nujol mulls and were taken between KBr disks using a Nicolet iS10 FT-IR spectrometer. Melting points were determined using an OptiMelt automated melting point system. UV−visible spectra were collected in hexane and determined with a Varian Cary 50 UV-visible spectrophotometer using a Schlenk-adapted 1 cm quartz cell. Elemental analyses were determined at the Microanalytical facility at University of California, Berkeley. All reagents were acquired from commercial sources and used without further purification. $Cp_2TiH_3AlC(SiMe_3)_3$ and $K[H_3AlC(SiMe_3)_3]$,[1] $KCp^‡$,[2] $UI_3(1,4\text{-dioxane})_{1.5}$,[3] $ThCl_4(DME)_2$[4] and KC_8[5] were synthesized according to previous literature procedures.

Synthesis of $Cp^‡_2ThCl_2$. A modified version of a literature procedure was used.[6] A slurry of $KCp^‡$ (3.28 g, 14.4 mmol) and $ThCl_4(DME)_2$ (4.00 g, 7.22 mmol) in THF (60 ml) was stirred for 2 days. Solvent was removed *in vacuo*, and the residue was extracted with *n*-hexane (2 x 40 ml) and filtered. The solution was concentrated until saturation and colorless crystals formed upon storage at -40 °C overnight (3.44 g, 72% yield). ^1H-NMR data was consistent with reported values.

Synthesis of $Cp^‡_2ThClH_3AlC(SiMe_3)_3$ (2). A solution of $K[H_3AlC(SiMe_3)_3]$ (0.64 g, 2.1 mmol) in toluene (~25 ml) was added to a solution of $Cp^‡_2ThCl_2$ (1.00 g, 1.52 mmol) in toluene (25 ml). The reaction mixture was stirred for 1 h, and the solvent was removed *in vacuo*. The white residue was extracted into *n*-hexane (30 ml) and filtered through Celite®. The solvent was removed *in vacuo* and a ^1H-NMR spectrum of the resulting white solid

was collected to determine the relative amount of $Cp^{‡}_2Th(H)H_3AlC(SiMe_3)_3$ based on the observed integrations. The residue was dissolved in toluene (10 ml) and a stoichiometric amount of chlorotrimethylsilane relative to the $Cp^{‡}_2Th(H)H_3AlC(SiMe_3)_3$ was added. The reaction was stirred overnight. The solvent was removed *in vacuo* and the resulting white solid was found to be analytically pure and used directly for subsequent reactions (1.20 g, 89% yield). X-ray quality crystals were grown from a HMDSO solution stored at room temperature for 4 days. ^{1}H NMR (500 MHz, C_6D_6, 293 K) δ 6.44 (m, 2H, Cp*H*), 6.29 (m, 4H, Cp*H*), 4.83 (s, 3H, Th-*H*-Al), 1.51 (s, 18H, C-C*H₃*), 1.37 (s, 18H, C-C*H₃*), 0.38 (s, 27H, Si-C*H₃*). ^{13}C{^{1}H} NMR (126 MHz, C_6D_6, 293 K) δ 150.93 (Cp), 149.89 (Cp), 115.40 (Cp), 115.03 (Cp), 113.66 (Cp). 34.24 (*C*-CH_3), 33.63 (*C*-CH_3), 32.69 (C-*C*H_3), 31.93 (C-*C*H_3), 5.36 (Si-*C*H_3). ^{27}Al NMR (104 MHz, C_6D_6, 293 K) no resonances observed. MP: dec > 177°C. FT-IR (KBr, Nujol, cm^{-1}): 1710 (m, Al-H stretch), 1599 (w, Al-H stretch). Anal calcd. (%) for $C_{36}H_{72}AlSi_3ThCl$: C, 48.93; H, 8.21 Found: C, 48.55; H, 8.36.

Synthesis of $Cp^{‡}_2Th(H)H_3AlC(SiMe_3)_3$ (3). A solution of $K[H_3AlC(SiMe_3)_3]$ (0.50 g, 1.7 mmol) in toluene (15 ml) was added to a solution of $Cp^{‡}_2ThCl_2$ (0.50 g, 0.76 mmol) in toluene (15 ml). The reaction mixture was refluxed for 2 days, and the solvent was removed *in vacuo*. The residue was extracted into *n*-hexane (30 ml) and filtered through Celite®, and the solvent was removed *in vacuo*. The residue was extracted into 10 ml of HMDSO, and concentrated to saturation (~2 ml). The solution was stored at -40 °C overnight to afford colorless crystals of the desired product (0.32 g, 50.% yield). ^{1}H NMR (500 MHz, C_6D_6, 293 K) δ 15.11 (s, Th*H*), 5.85 (m, 2H, Cp*H*), 5.71 (m, 4H, Cp*H*), 3.49 (s, 3H, Th-*H*-Al), 1.59 (s, 18H, C-C*H₃*), 1.36 (s, 18H, C-C*H₃*), 0.34 (s, 27H, Si-C*H₃*). ^{13}C{^{1}H} NMR (126 MHz, C_6D_6, 293 K) δ 150.12 (Cp), 146.94 (Cp), 110.48 (Cp), 110.29 (Cp), 107.94 (Cp). 33.28 (C-*C*H_3), 33.24 (*C*-CH_3), 33.13 (C-*C*H_3), 32.74 (*C*-CH_3), 5.11 (Si-*C*H_3). ^{27}Al NMR (104 MHz, C_6D_6, 293 K) no resonances observed. MP: dec > 173°C. FT-IR (KBr, Nujol, cm^{-1}): 1682 (m, Al-H stretch), 1602 (w, Al-H stretch). Anal calcd. (%) for $C_{36}H_{72}AlSi_3Th$: C, 50.91; H, 8.66 Found: C, 50.54; H, 8.43.

Synthesis of $Cp^{‡}_2ThH_3AlC(SiMe_3)_3$ (4). A solution of $Cp^{‡}_2ThClH_3AlC(SiMe_3)_3$ (0.35 g, 0.40 mmol) in *n*-hexane (6 ml) was added to a stirring suspension of KC_8 (0.083 mg, 0.60 mmol) in *n*-hexane (4 ml) to produce a dark purple solution. The reaction was stirred for 24 h and an additional 1.5 equivalents of KC_8 (0.083 mg, 0.60 mmol) were added to the

stirring solution. The reaction was stirred for 4 additional days, and the solvent was removed *in vacuo*. The dark purple residue was extracted into HMDSO and filtered through Celite®. The purple solution was concentrated to saturation, and storage of the solution at -40 °C overnight afforded the desired product as dark purple crystals (0.116 g of a 1:0.15:0.5 mixture of **4**:**3**:**2** as determined by ^{1}H NMR, 28% yield). X-ray quality crystals could be grown upon recrystallization from HMDSO found to be 1:0.12 mixtures of **4:3**. ^{1}H NMR (600 MHz, C_6D_6, 293 K) δ 5.81 (s, br), 0.56 (s, br). UV-vis, [nm, ε (M^{-1} cm^{-1})] 360., 1694; 440., 730.; 520, 3999; 585, 2150.; 640., 2482. MP: dec > 190.5 °C. FT-IR (KBr, Nujol, cm^{-1}): 1674 (m, Al-H stretch). Anal calcd. (%) for $C_{36}H_{72}AlSi_3Th$: C, 50.98; H, 8.56 Found: C, 50.68; H, 8.32. μ_{eff} = 1.83 μ_B at 297.1 K.

Synthesis of [Cp‡$_2$UI]$_2$. An adaptation of literature procedure for the synthesis of [Cp‡$_2$UCl]$_2$ was used.[7] UI_3(1,4-dioxane)$_{1.5}$ was dissolved in THF and stirred for 30 minutes, then the solvent was removed *in vacuo*. The dark blue solid was dissolved in THF, and a solid suspension of KCp‡ in THF was added. The reaction mixture was stirred for 16 hours resulting in a dark green solution. The solvent was removed *in vacuo*. The residue was extracted with hexane, and the green solution was filtered through Celite®. The solvent was removed *in vacuo* and the crude green solid was used without further characterization. The IR spectrum was consistent with previous literature reports.[8]

Synthesis of Cp‡$_2$UH$_3$AlC(SiMe$_3$)$_3$ (5). A solution of K[$H_3AlC(SiMe_3)_3$] (0.12 g, 0.40 mmol) in toluene (4 ml) was added to a solution of [Cp‡$_2$UI]$_2$ (0.25 g, 0.35 mmol) in toluene (6 ml) to produce a brown solution. The reaction mixture was stirred overnight, and the solvent was removed *in vacuo*. The brown residue was triturated with *n*-hexane and extracted with HMDSO (10 ml). The brown solution was filtered through Celite®, and concentrated until saturated (~ 4 ml). Storage of the solution at -40 °C overnight afforded the desired product as dark yellow crystals (0.12 g). A second crop was isolated upon further concentration of the remaining solution (0.033 g, 51% total yield). ^{1}H NMR (500 MHz, C_6D_6, 293 K) δ 40.93 (s, br), 2.15 (s, br), -14.03 (s, br), -32.28 (s, br). UV-vis, [nm, ε (M^{-1} cm^{-1}) 337, 2282; 411, 816; 475, 299. MP: dec > 185 °C. FT-IR (KBr, Nujol, cm^{-1}): 1580 (w, Al-H stretch). Anal calcd. (%) for $C_{36}H_{72}AlSi_3U$: C, 50.62; H, 8.50 Found: C, 50.46; H, 8.53. μ_{eff} = 2.88 μ_B at 296.8 K.

B. NMR Spectra

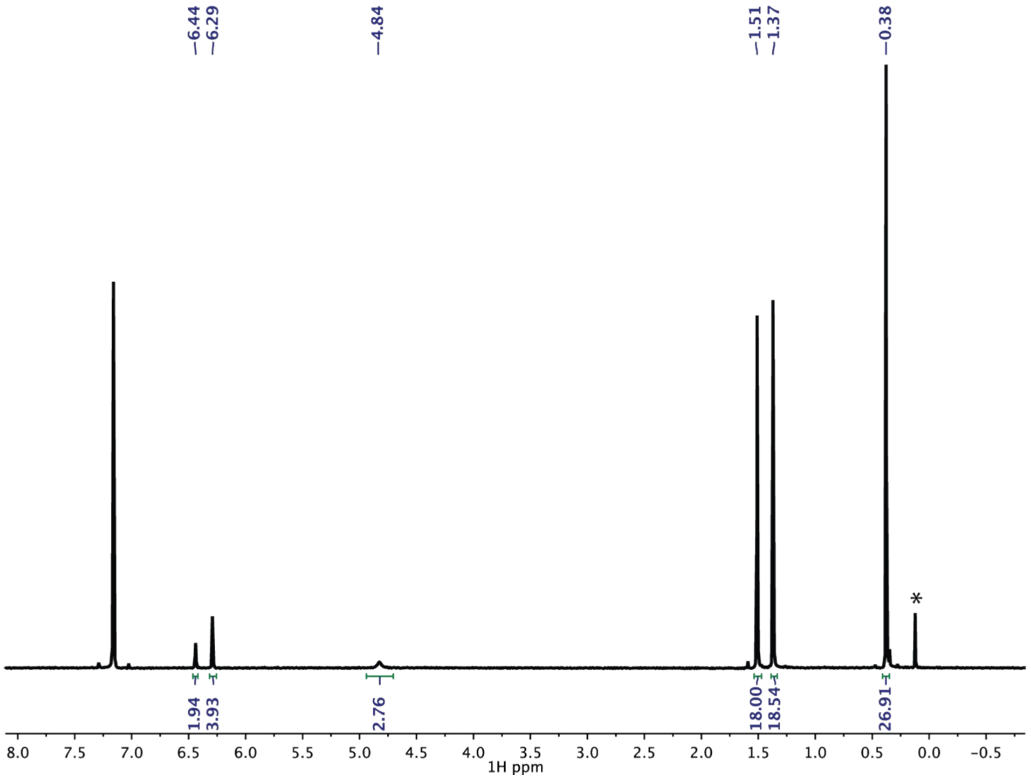

Figure S1. ^{1}H NMR Spectrum of **2** in C_6D_6 at 293 K (*= residual HMDSO)

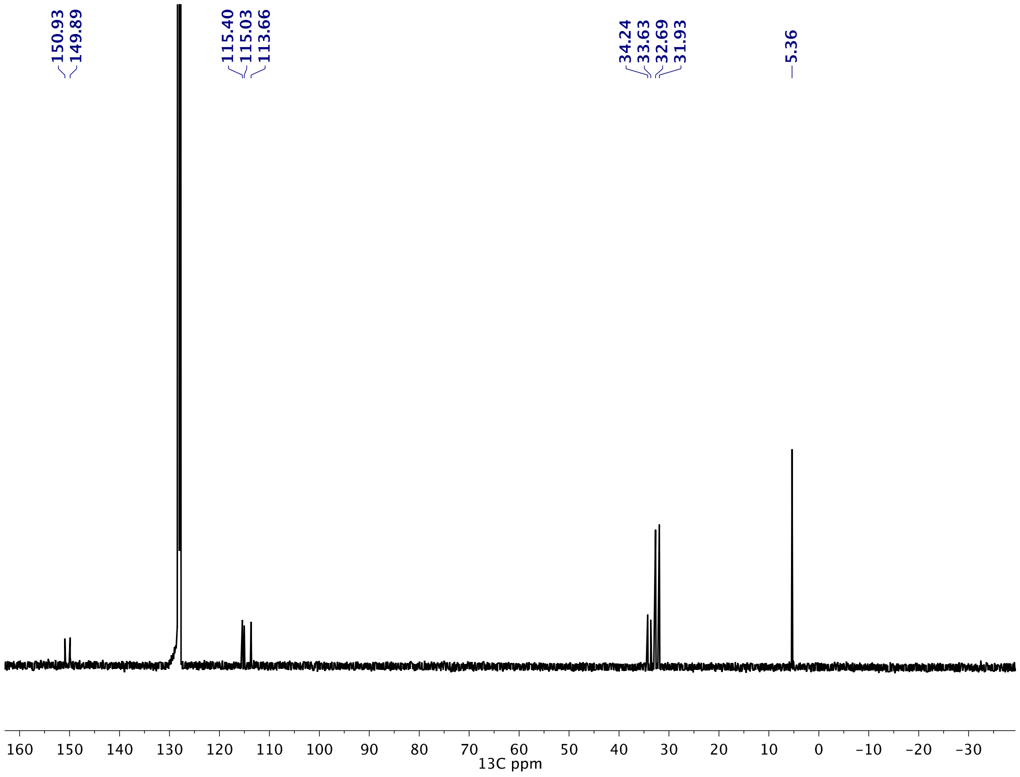

Figure S2. ^{13}C NMR Spectrum of **2** in C_6D_6 at 293 K.

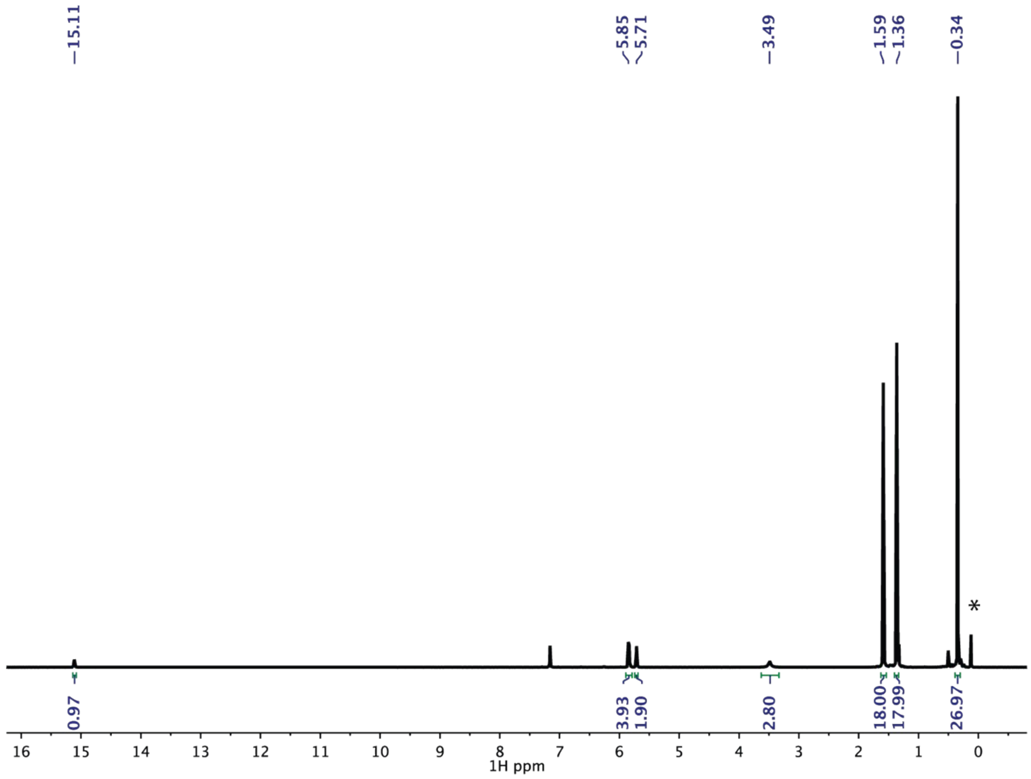

Figure S3. ^{1}H NMR Spectrum of **3** in C_6D_6 at 293 K (*= residual HMDSO).

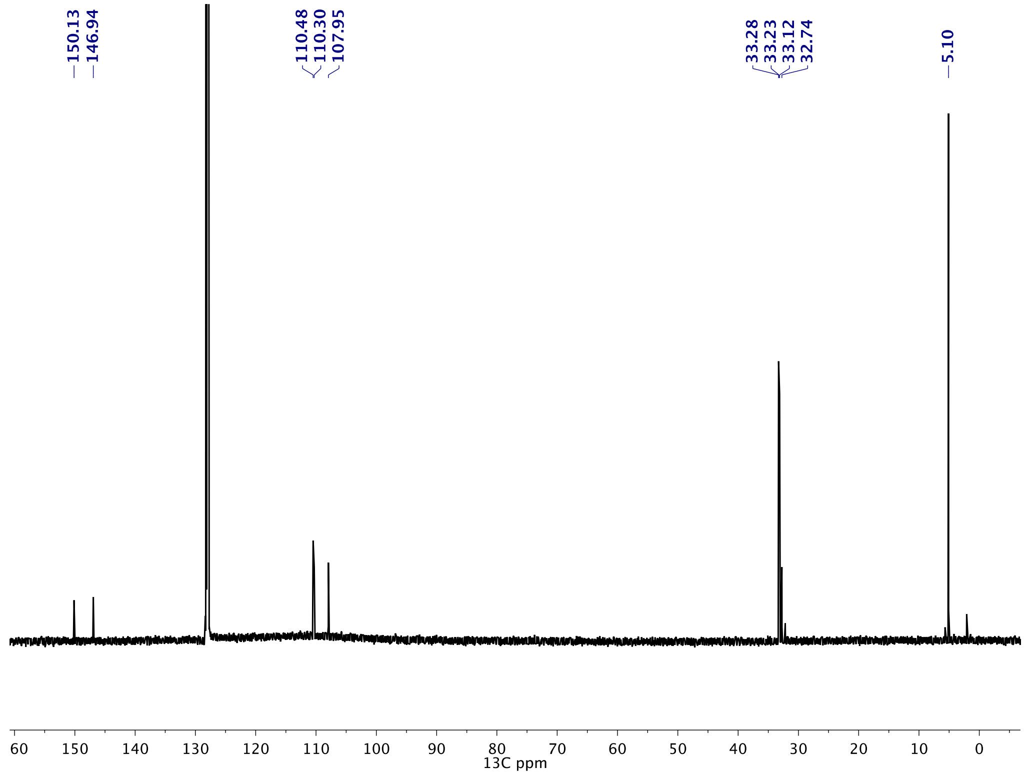

Figure S4. ^{13}C NMR Spectrum of **3** in C_6D_6 at 293 K.

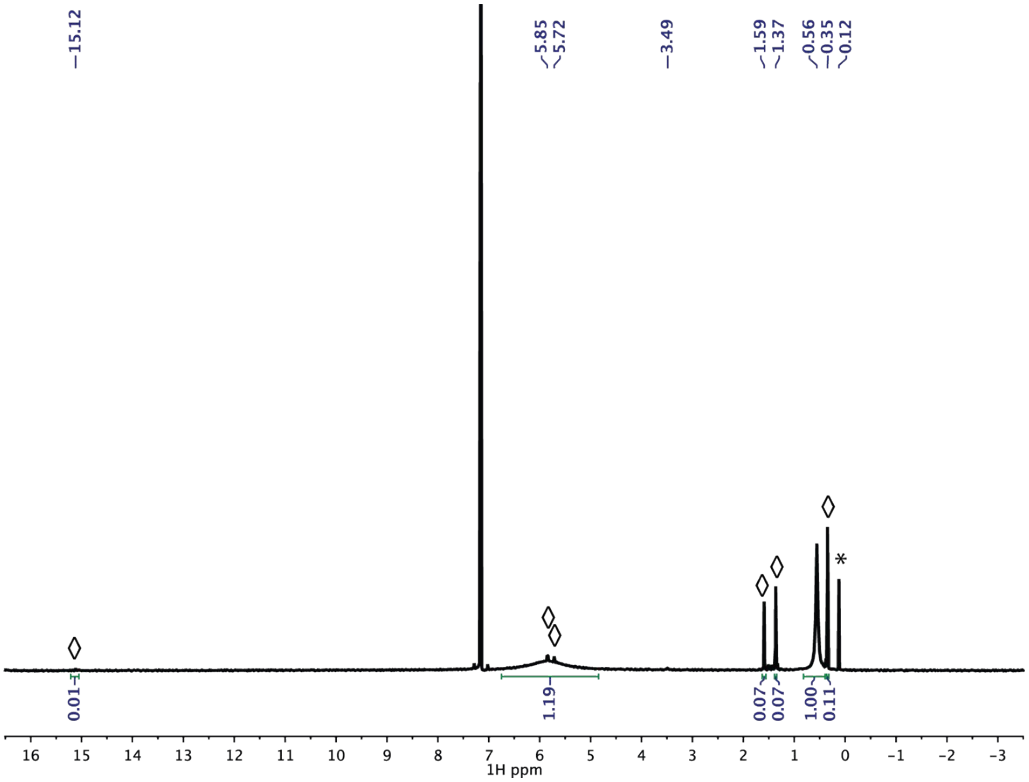

Figure S5. ^{1}H NMR Spectrum of **4** in C_6D_6 at 293 K (◊=**3**, ~11%, *= residual HMDSO).

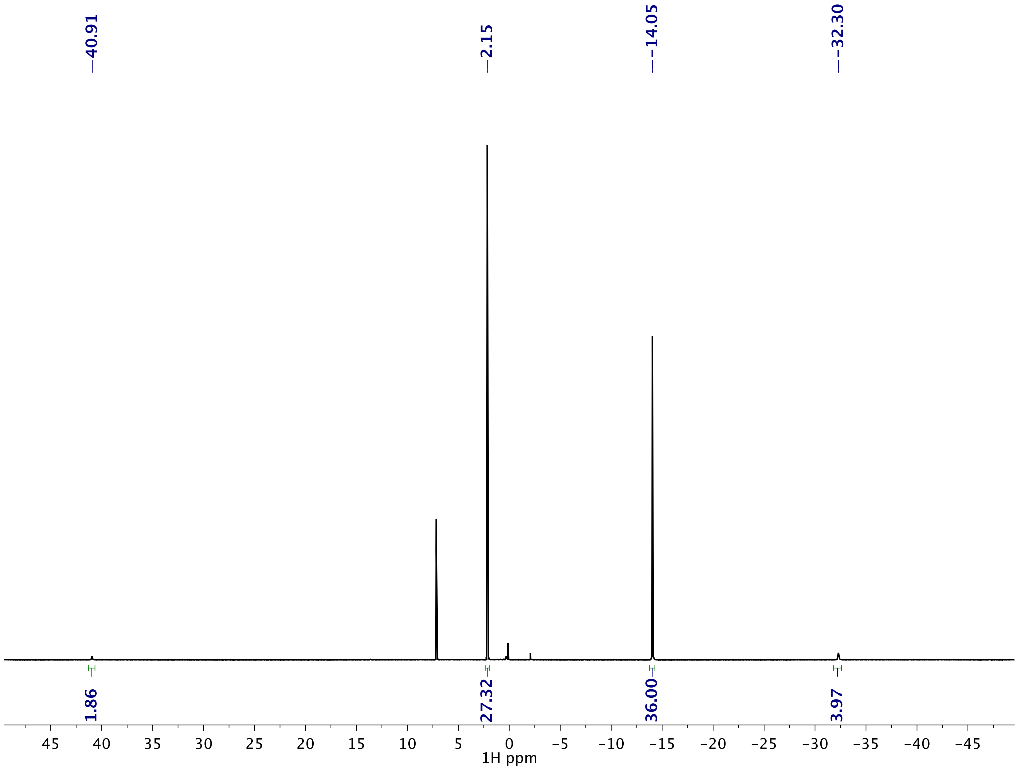

Figure S6. ^{1}H NMR Spectrum of **5** in C_6D_6 at 293 K.

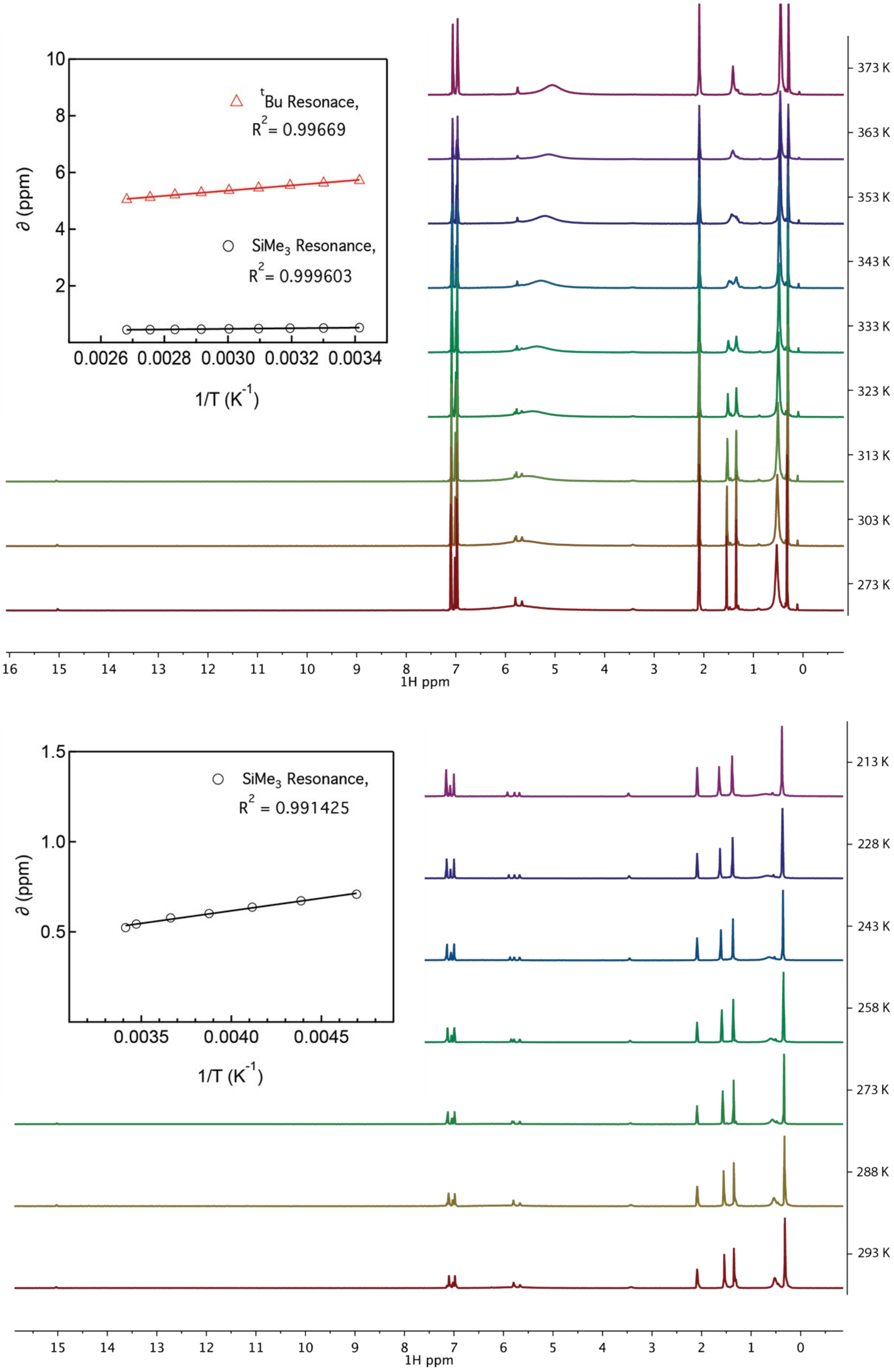

Figure S7. ^{1}H NMR Spectrum of **4** in d^{8}-toluene at varying temperatures. Paramagnetic resonance shows linear dependence with1/T, following the Curie law.

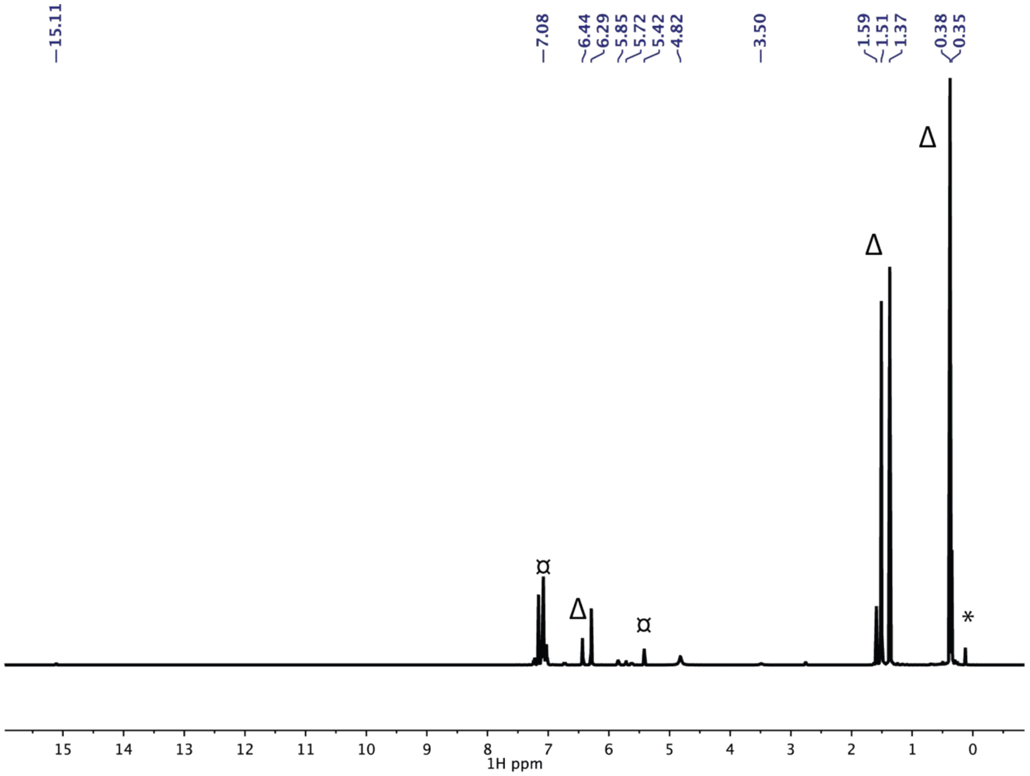

Figure S8. ^{1}H NMR spectrum of products resulting from the reaction of **3** with triphenylchloromethane in C_6D_6 at 293 K (Δ= **4**, ¤= triphenylmethane, *= residual HMDSO).

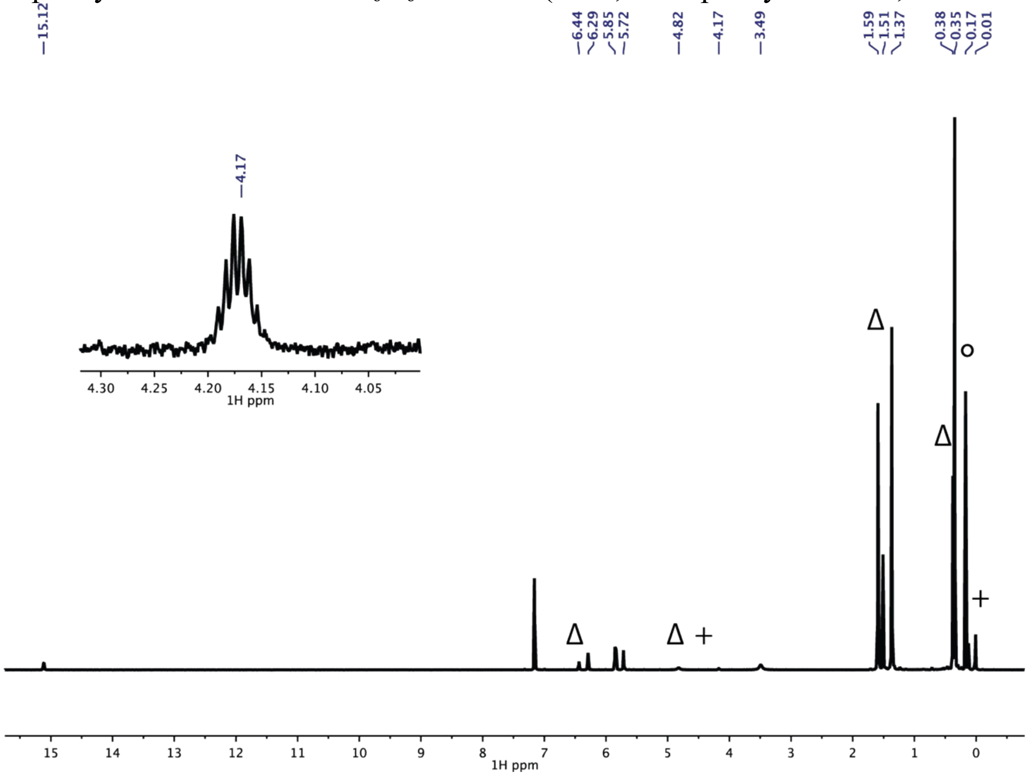

Figure S9. Representative ^{1}H NMR spectrum of products resulting from the reaction of **3** with trimethylchlorosilane in C_6D_6 at 293 K (reaction had not gone to completion, Δ= **4**, += trimethylsilane, °= trimethylchlorosilane). Inset shows dectet resonance from silane generated.[9]

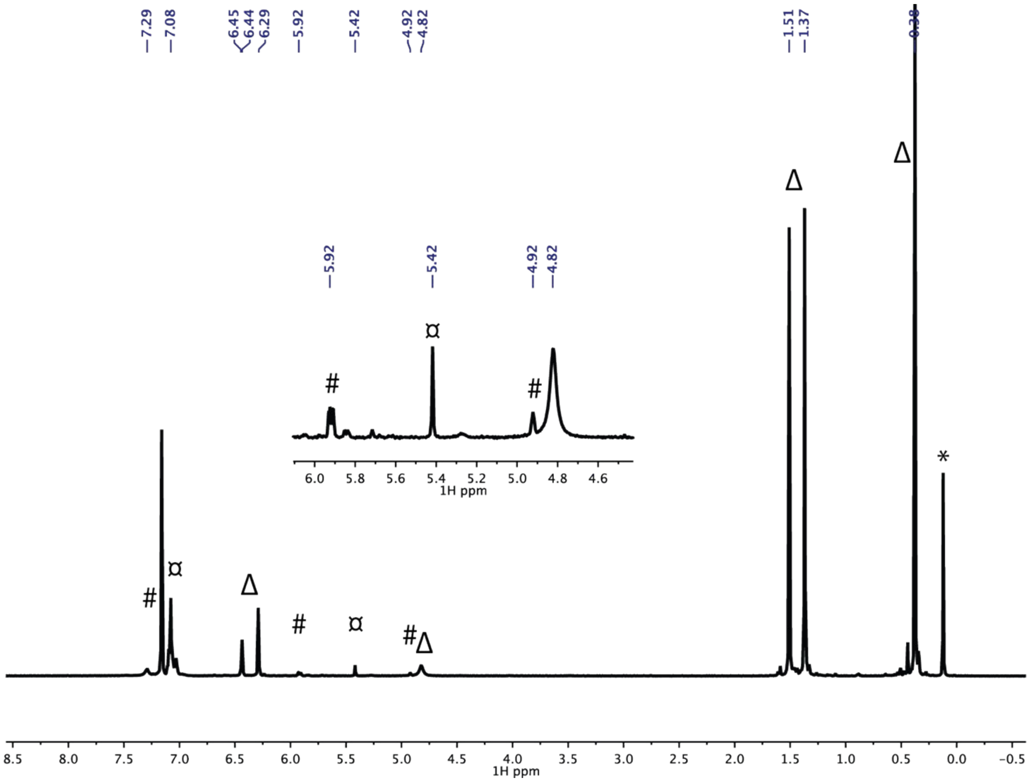

Figure S10. ^{1}H NMR spectrum of products resulting from the reaction of **4** with triphenylchloromethane in C_6D_6 at 293 K (Δ= **4**, #= Gomberg's dimer, ¤= triphenylmethane, *= residual HMDSO). Inset shows diagnostic resonances corresponding to triphenylmethane and Gomberg's dimer.[10]

C. Infrared Spectra

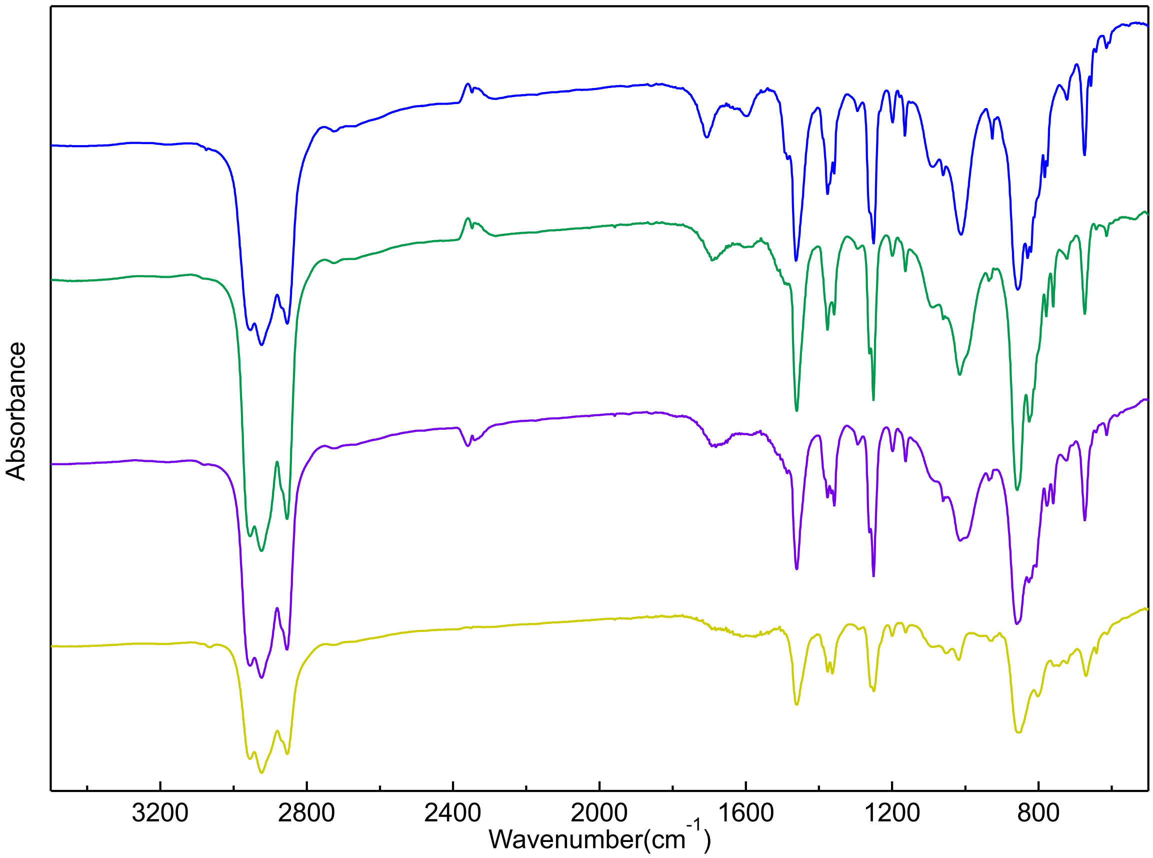

Figure S11: Infrared spectrum of **2** (blue), **3** (green), **4** (purple), **5** (yellow). Compound **2** (KBr, Nujol, cm^{-1}): 1708 (m), 1597 m), 1463 (s), 1377 (m), 1251 (s), 1199 (w), 1166 (w), 1012 (m, br), 927 (w), 857 (s, br), 645 (m). Compound **3** (KBr, Nujol, cm^{-1}): 1693 (m), 1461 (s), 1377 (m), 1252 (s), 1200. (w), 1164 (w), 1016 (m, br), 859 (s, br), 674 (m). Compound **4** (KBr, Nujol, cm^{-1}): 1683 (m), 1462 (s), 1377 (s), 1252 (s), 1199(w), 1164 (w), 1015 (m, br), 860. (s, br), 674 (m). Compound **5** (KBr, Nujol, cm^{-1}): 1574 (m), 1462 (s), 1377 (m), 1251 (s), 1200. (w), 1164 (w), 1019 (m, br), 853 (s, br), 671 (m).

D. UV-Visible Spectra

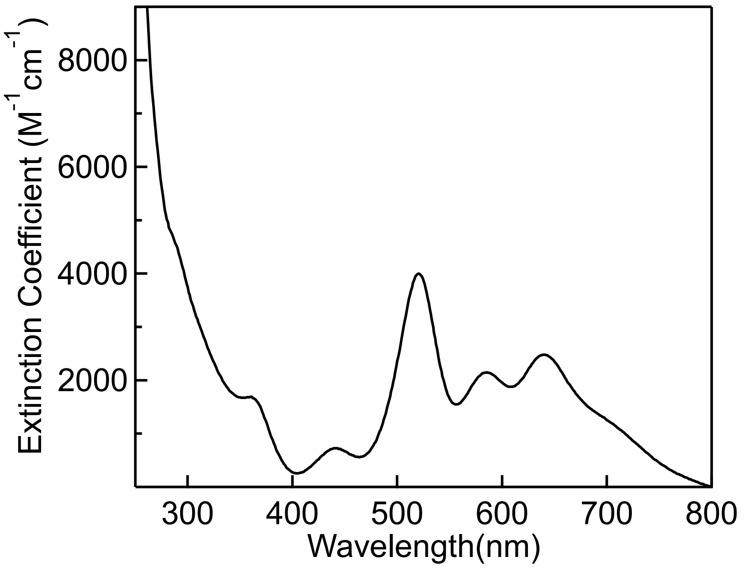

Figure S12: UV-Visible spectrum of **4** in hexane at 295 K.

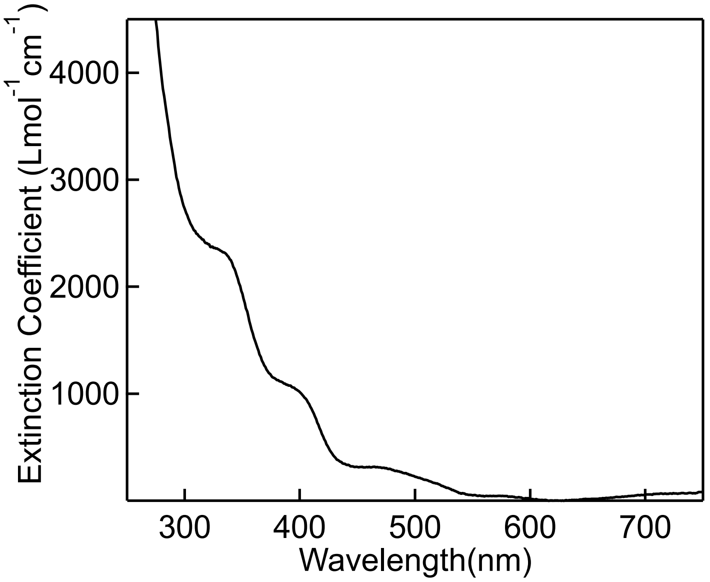

Figure S13: UV-Visible spectrum of **5** in hexane at 295 K.

E. Crystallographic Details

X-ray diffraction data for Cp‡$_2$ThClH$_3$AlC(SiMe$_3$)$_3$ (**2**) and Cp‡$_2$UH$_3$AlC(SiMe$_3$)$_3$ (**5**) were collected at CheXray, Berkeley, CA, using a Bruker APEX II QUAZAR instrument outfitted with a microfocus Mo-Kα radiation source (λ = 0.71073 Å). Diffraction data for Cp‡$_2$Th(H)H$_3$AlC(SiMe$_3$)$_3$ (**3**) and Cp‡$_2$ThH$_3$AlC(SiMe$_3$)$_3$ (**4**) were obtained at the Advanced Light Source (ALS), Lawrence Berkeley National Laboratory, Berkeley, CA, station 11.3.1, using a silicon-monochromated beam of 16 keV (λ = 0.7749 Å) radiation. All data collections were conducted at 100 K. Absorption corrections were performed by a multiscan method, utilizing either Bruker AXS SADABS or TWINABS (for **3**) as appropriate.[11] Bruker APEX3 software was used for the data collection, and Bruker SAINT software for the cell refinement and data reduction procedures.[12] Initial structure solutions were found using direct methods (SHELXT) and were subjected to full-matrix refinements against F^2 by SHELXL-2014.[13] All non-hydrogen atoms in all structures were refined anisotropically. Hydrogen atoms bound to or bridging between metal centers could be explicitly located in all structures and were refined isotropically with no positional restraints.

Table S1. Crystallographic details for compounds **2-5.**

	**2**	**3**	**4**	**5**
Chemical formula	$C_{36}H_{72}AlSi_3ThCl$	$C_{36}H_{73}AlSi_3Th$	$C_{36}H_{72}AlSi_3Th$	$C_{36}H_{72}AlSi_3U$
Formula weight	883.67	849.23	848.22	854.21
Color, habit	Colorless, block	Colorless, plate	Purple, tablet	Brown, blocks
Temperature (K)	100(2)	100(2)	100(2)	100(2)
Crystal system	Monoclinic	Monoclinic	Monoclinic	Triclinic
Space group	P 2_1/n	P 2_1/c	P 2_1/m	P -1
a (Å)	14.6013(10	18.541(16)	12.933(8)	10.4534(5)
b (Å)	16.3421(10)	12.528(10)	12.816(8)	12.8540(6)
c (Å)	18.0401(11)	105.644(9)	13.179(8)	17.8476(8)
α (°)	90	90	90	72.090(2)
β (°)	93.323(3)	105.644(9)	94.460(4)	79.893(2)
γ (°)	90	90	90	66.898(2)
V (Å^3)	4297.4(5)	4243(6)	2178(2)	2094.82(17)
Z	4	4	2	2
Density (Mg m^{-3})	1.366	1.329	1.294	1.354
F(000)	1800	1736	866	870
Radiation Type	MoK$_α$	Synchrotron	Synchrotron	MoK$_α$
μ (mm^{-1})	3.658	1.962	1.912	4.002
Crystal size	0.20 x 0.20 x 0.10	0.04 x 0.03 x 0.01	0.09 x 0.06 x 0.02	0.2 x 0.2 x 0.2
Meas. Refl.	133659	35013	22006	58071
Indep. Refl.	7921	7699	5198	7694
R(int)	0.0542	0.1080	0.0346	0.0239
Final R indices	R = 0.0254	R = 0.0477	R = 0.0240	R = 0.0188
[I > 2σ(I)]	R$_w$ = 0.0561	R$_w$ = 0.0840	R$_w$ = 0.571	R$_w$ = 0.0437
Goodness-of-fit	1.142	1.034	1.059	1.040
Δρ$_{max}$, Δρ$_{min}$, (e Å^{-3})	2.709, -0.948	2.533, -1.962	1.431, -1.273	1.163, -0.911
CCDC	1822996	1822997	1822998	1822999

F. Electron Paramagnetic Resonance Spectra

EPR spectroscopy was performed in the CalEPR center in Department of Chemistry, University of California at Davis. CW-EPR experiments were performed on the Bruker Biospin EleXsys E500 spectrometer with a super high Q resonator (ER4122SHQE) in perpendicular mode. Cryogenic temperature was achieved by using an ESR900 liquid helium cryostat with a temperature controller (Oxford Instrument ITC503) and gas flow controller. All continuous-wave (CW) EPR spectra were recorded under slow-passage, non-saturating conditions. Spectrometer settings were as following: conversion time = 30 ms, modulation frequency = 100 kHz, and parameters in the corresponding figure legends. Pulsed Q-band electron spin-echo detected field swept EPR (ESE-FS) was recorded on the Bruker Biospin EleXsys 580 spectrometer equipped with a R.A. Isaacson cylindrical TE_{011} resonator. Standard Hahn-echo pulse sequence (π/2-τ-π-τ-echo) was used for ESE-FS experiments. Simulations of EPR spectra were performed in Matlab 2014a with the EasySpin 5.1.10 toolbox.[14]

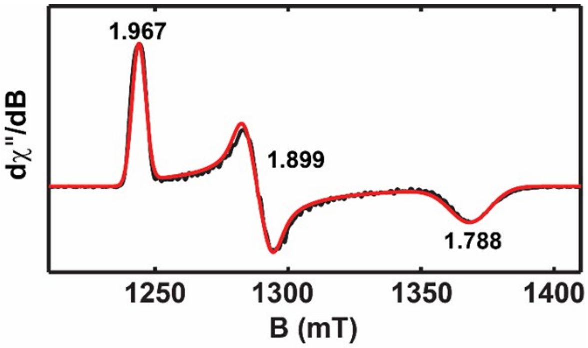

Figure S14. Q-band (34 GHz) pseudo-modulated free induction decay detected field-swept EPR spectrum of **4** (black trace) and simulation (red trace). Conditions: temperature, 15 K; time interval between the first (π/2) and the second (π) microwave pulse (τ) = 300 ns; modulation amplitude, 0.5 mT.

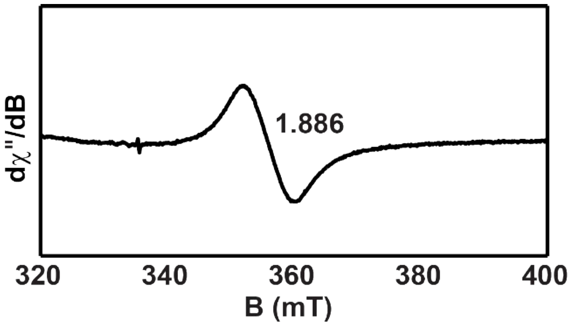

Figure S15. X-band (9 GHz) CW-EPR of **4** in recorded in toluene solution (230 K). Conditions: microwave power, 0.5 mW; modulation amplitude, 0.5 mT.

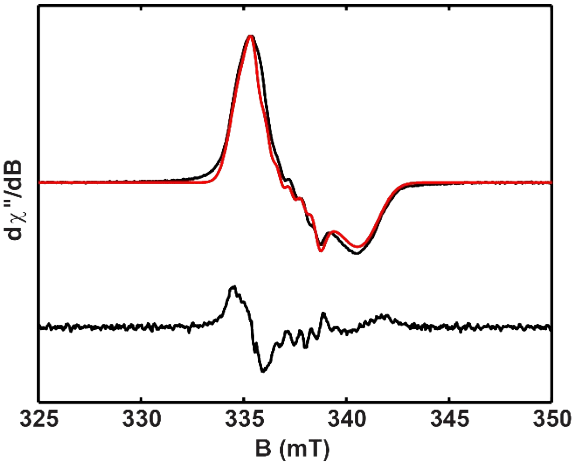

Figure S16. The numeric 2nd derivative (bottom trace) of the CW-EPR of **6** (top trace) showing the hyperfine splitting from ^{27}Al at different field positions. Conditions: temperature, 50 K; microwave power, 0.02 mW; modulation amplitude, 0.2 mT.

Note: the hyperfine splittings observed in **6** are not caused by the HFI from the bridging hydrides, since similar patterns can be observed with a deuteride isotopologue. HFI from ^{1}H will be analyzed further in a pulse EPR study.

G. Computational Details

All the structures reported in this study were fully optimized with the Becke's 3-parameter hybrid functional[15] combined with the non-local correlation functional provided by Perdew/Wang (denoted as B3PW91).[16] The basis sets used for titanium, thorium, uranium as well as silicon and aluminum atoms were the Stuttgart-Dresden small core ECP in combination with its adapted basis set.[17,18] For the rest of the atoms the 6-31G(d,p) basis set was used.[19-21] In all computations no constrains were imposed on the geometry. All stationary points have been identified for minima (number of imaginary frequencies Nimag=0). The vibrational modes and the corresponding frequencies are based on a harmonic force field. The GAUSSIAN09 program suite was used in all calculations.[22] Natural Bonding Orbital (NBO)[23,24] analyses were carried out using the associated module in the Gaussian package. Atoms in Molecule (AIM)[25,26] analysis was also performed using the Multiwfn code.[27]

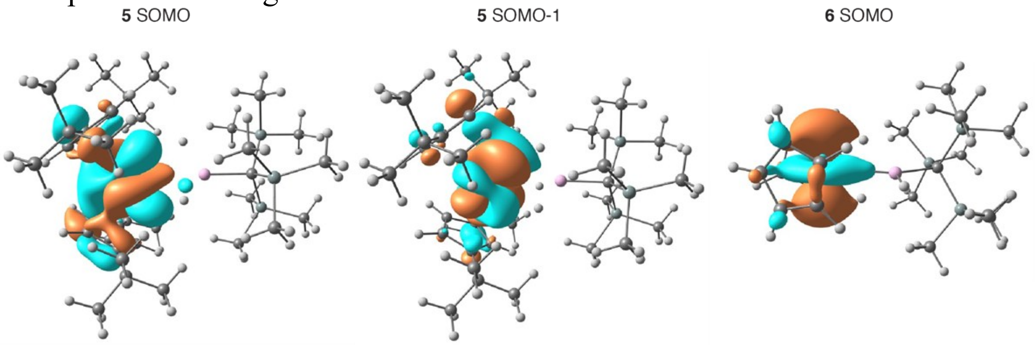

Figure S17: Valence orbitals for **5** and **6**. Aluminum atoms shown in pink.

Cartesian coordinates of all optimized structures

Ti-Al complex

22	4.884935000	2.969762000	8.301599000
14	0.044968000	4.253662000	5.799760000
14	2.404326000	3.414548000	3.880168000
14	2.151476000	6.439026000	4.848510000
13	2.934930000	4.459858000	6.986733000
6	6.539282000	2.218634000	6.821603000
1	7.207456000	2.929315000	6.353304000
6	5.276722000	1.800621000	6.321266000
1	4.825134000	2.127190000	5.397631000
6	1.874111000	4.578558000	5.297090000
6	4.910766000	4.949478000	9.612149000
1	4.803210000	5.918337000	9.145119000
6	4.724686000	0.864354000	7.233589000
1	3.767280000	0.370170000	7.139256000
6	5.628982000	0.719093000	8.305225000
1	5.487523000	0.092421000	9.176294000
6	5.802705000	3.017907000	10.454165000
1	6.509994000	2.260819000	10.764096000
6	6.122773000	4.252309000	9.829640000

1	7.114094000	4.601660000	9.571092000
6	4.141005000	3.852474000	3.218954000
1	4.888610000	3.939023000	4.014077000
1	4.475161000	3.072170000	2.525529000
1	4.138701000	4.798088000	2.668099000
6	6.755696000	1.547567000	8.043292000
1	7.625813000	1.656120000	8.676886000
6	3.846726000	4.141947000	10.083495000
1	2.798431000	4.400993000	10.044068000
6	4.394781000	2.939797000	10.596340000
1	3.838461000	2.118603000	11.029158000
6	3.980284000	6.928304000	5.097116000
1	4.293655000	6.839318000	6.143738000
1	4.665640000	6.334197000	4.487344000
1	4.106935000	7.980322000	4.815277000
6	-0.356074000	4.840234000	7.569036000
1	-0.131552000	5.895013000	7.738684000
1	-1.426683000	4.683430000	7.747823000
1	0.186580000	4.259509000	8.323830000
6	-0.416285000	2.405432000	5.825396000
1	0.271238000	1.814500000	6.437896000
1	-1.415220000	2.315792000	6.268056000
1	-0.458278000	1.955110000	4.830111000
6	-1.216912000	5.077460000	4.633482000
1	-1.161403000	6.169166000	4.649047000
1	-1.086766000	4.749998000	3.597985000
1	-2.228504000	4.794434000	4.947045000
6	1.251459000	3.431411000	2.362450000
1	1.662150000	2.752873000	1.605175000
1	0.249507000	3.068505000	2.612223000
1	1.151765000	4.418900000	1.906388000
6	1.179125000	7.658369000	5.940747000
1	1.448006000	7.545204000	6.994270000
1	1.446365000	8.676240000	5.633012000
1	0.093912000	7.563905000	5.849869000
6	1.714301000	6.888239000	3.050301000
1	2.354786000	6.388268000	2.318410000
1	0.673437000	6.651519000	2.812220000
1	1.848723000	7.968592000	2.921616000
6	2.449485000	1.575383000	4.408288000
1	2.559834000	1.444911000	5.487031000
1	1.526267000	1.069851000	4.112194000
1	3.277060000	1.055207000	3.913469000
1	4.653193000	4.331834000	7.015762000
1	3.030805000	2.963438000	7.844500000
1	2.568582000	5.660275000	7.968718000

Th-Al complex

6	6.523079000	10.010201000	7.769744000
1	6.209367000	9.196430000	7.126297000
6	8.745158000	13.756874000	9.823141000
1	7.944515000	13.781877000	10.569341000
1	9.165290000	14.767026000	9.758089000
1	9.524611000	13.081295000	10.189746000
6	9.537756000	7.195677000	8.051768000
6	9.263112000	9.765254000	14.263226000
6	6.009454000	10.292913000	9.064715000
6	7.465049000	11.009889000	7.423477000
1	7.989463000	11.069187000	6.477731000
6	8.227865000	13.310551000	8.448282000
6	11.232971000	8.795246000	8.207458000
6	4.621732000	10.035112000	11.165283000
1	5.486438000	9.709190000	11.749977000
1	3.727237000	9.561254000	11.584195000
1	4.509538000	11.116673000	11.292431000
6	10.694203000	7.610161000	8.779791000
1	11.132140000	7.067296000	9.607782000
6	7.582835000	11.929423000	8.502480000
6	12.601649000	9.401353000	8.496820000
6	4.775937000	9.649327000	9.689496000
6	9.342967000	8.172562000	7.039133000
1	8.556321000	8.171199000	6.294278000
6	7.493210000	5.831497000	7.469904000
1	6.816334000	6.557772000	7.930904000
1	7.030271000	4.841793000	7.550379000
1	7.580276000	6.067051000	6.404153000
6	6.691045000	11.456632000	9.516850000
1	6.512491000	11.949975000	10.463365000
6	8.678980000	5.409679000	9.628695000
1	9.632373000	5.380781000	10.165485000
1	8.239705000	4.407000000	9.682225000
1	8.011891000	6.098202000	10.160101000
6	13.136069000	8.944590000	9.860377000
1	12.455413000	9.221564000	10.670728000
1	14.107877000	9.409475000	10.058598000
1	13.278769000	7.859588000	9.892982000
6	7.140172000	14.303418000	7.981860000
1	6.753431000	14.025956000	6.995791000
1	7.548050000	15.319179000	7.915985000
1	6.297113000	14.319498000	8.680097000
6	10.364776000	9.151696000	7.141064000
1	10.487785000	9.999294000	6.476787000

6	12.567314000	10.935995000	8.457912000
1	12.228505000	11.306321000	7.484937000
1	13.569235000	11.343795000	8.634362000
1	11.895536000	11.338430000	9.224427000
6	4.808563000	8.118238000	9.577343000
1	4.883726000	7.791294000	8.535269000
1	3.888735000	7.687800000	9.989844000
1	5.658886000	7.701971000	10.128308000
6	9.388382000	13.351490000	7.444949000
1	10.188663000	12.663788000	7.736392000
1	9.812314000	14.360804000	7.401370000
1	9.060638000	13.092009000	6.432811000
6	3.548161000	10.176181000	8.914060000
1	3.491219000	11.268306000	8.966087000
1	2.623193000	9.764956000	9.335547000
1	3.596065000	9.892836000	7.857677000
6	8.861359000	5.833710000	8.163184000
6	13.566921000	8.899931000	7.400577000
1	13.609193000	7.805983000	7.388636000
1	14.580207000	9.279448000	7.578238000
1	13.246409000	9.234101000	6.408703000
6	9.772429000	4.804234000	7.459821000
1	9.923698000	5.069958000	6.408507000
1	9.326047000	3.803409000	7.499179000
1	10.755669000	4.757264000	7.938654000
13	9.043820000	9.675994000	12.314445000
14	11.159756000	9.793393000	14.582716000
14	8.405096000	11.375656000	14.866750000
14	8.425030000	8.180467000	14.958522000
90	8.739298000	9.545187000	9.383465000
6	6.763282000	11.678951000	13.953459000
1	6.331860000	12.626304000	14.297598000
1	6.021303000	10.894797000	14.118072000
1	6.925546000	11.771270000	12.873745000
6	12.060834000	10.967996000	13.383672000
1	13.142035000	10.827979000	13.498134000
1	11.839020000	12.019881000	13.575854000
1	11.818047000	10.762744000	12.335411000
6	8.770055000	6.663422000	13.859670000
1	8.264268000	5.792197000	14.292269000
1	9.832070000	6.423637000	13.772902000
1	8.369323000	6.799449000	12.848870000
6	11.613310000	10.343933000	16.346435000
1	12.701284000	10.293012000	16.469149000
1	11.161207000	9.698443000	17.104368000
1	11.307419000	11.373707000	16.552557000

6	6.525706000	8.301631000	14.998907000
1	6.113450000	8.478293000	14.000549000
1	6.146036000	9.077672000	15.669004000
1	6.129041000	7.342020000	15.350700000
6	9.424509000	12.942851000	14.509428000
1	9.662300000	13.035912000	13.445122000
1	10.358418000	12.997537000	15.075453000
1	8.820642000	13.813937000	14.789547000
6	8.980225000	7.749521000	16.726837000
1	10.046357000	7.512910000	16.787698000
1	8.427400000	6.867334000	17.069639000
1	8.773955000	8.564099000	17.427010000
6	11.976744000	8.096512000	14.310435000
1	13.058620000	8.209003000	14.446629000
1	11.808715000	7.727727000	13.293826000
1	11.635175000	7.332025000	15.013642000
6	8.040618000	11.380830000	16.733997000
1	8.952196000	11.256183000	17.325372000
1	7.336715000	10.597761000	17.029298000
1	7.594898000	12.344104000	17.007564000
1	7.715214000	9.041880000	11.502060000
1	10.054350000	8.806632000	11.321143000
1	9.130427000	10.972313000	11.267107000

U-Al complex

6	5.058473000	9.567181000	3.550640000
6	3.376115000	7.958109000	1.401196000
1	2.403775000	7.923281000	0.895780000
1	3.504259000	7.003054000	1.922818000
1	4.150404000	8.030212000	0.634246000
6	1.919449000	9.057400000	3.785846000
1	1.717609000	9.860450000	4.500105000
1	2.077091000	8.130859000	4.346370000
1	1.018043000	8.925399000	3.176052000
6	2.920354000	10.987264000	1.646612000
1	3.621322000	11.203266000	0.835104000
1	2.871810000	11.867207000	2.294402000
1	1.929966000	10.849708000	1.197456000
6	6.386091000	9.672744000	0.655593000
1	7.239776000	10.092153000	0.110694000
1	5.479858000	9.953875000	0.112658000
1	6.479245000	8.582747000	0.627278000
6	6.363496000	12.221623000	2.336550000
1	6.538484000	12.704776000	3.302190000
1	5.401888000	12.573449000	1.952558000
1	7.143775000	12.569078000	1.649528000

6	8.174354000	9.855261000	3.062768000
1	8.320355000	8.769801000	3.057748000
1	8.391128000	10.220338000	4.068964000
1	8.920226000	10.283207000	2.382696000
6	4.088295000	9.382719000	6.554395000
1	3.068396000	9.081081000	6.306855000
1	4.056698000	9.928356000	7.504632000
1	4.680218000	8.475421000	6.719108000
6	6.567635000	10.971094000	5.984164000
1	7.206338000	10.095822000	6.138837000
1	6.382683000	11.420269000	6.966988000
1	7.124714000	11.700843000	5.390552000
6	3.867324000	12.089190000	5.114077000
1	4.280088000	12.783651000	4.376699000
1	3.881770000	12.590831000	6.088568000
1	2.820318000	11.905136000	4.855819000
13	5.619379000	7.717581000	3.943533000
14	3.382981000	9.420272000	2.624001000
14	6.434130000	10.322034000	2.444126000
14	4.889638000	10.486330000	5.225460000
1	6.703227000	7.331743000	5.163466000
1	4.519536000	6.547642000	4.418613000
1	6.347376000	6.767266000	2.818598000
6	7.243414000	3.967195000	2.048783000
1	6.928279000	4.542192000	1.186910000
6	8.457017000	4.159552000	2.756522000
6	8.483657000	3.175996000	3.788930000
1	9.294558000	3.006759000	4.488957000
6	7.321763000	2.370279000	3.675567000
1	7.092950000	1.513954000	4.297790000
6	6.536661000	2.855227000	2.596526000
6	9.601703000	5.092973000	2.379915000
6	5.305790000	2.205363000	1.975895000
6	9.203554000	6.019134000	1.223437000
1	10.031049000	6.694051000	0.979016000
1	8.332457000	6.629744000	1.480904000
1	8.965254000	5.447084000	0.320747000
6	10.801328000	4.233521000	1.931132000
1	10.527480000	3.597965000	1.083028000
1	11.148494000	3.581223000	2.738693000
1	11.639616000	4.870726000	1.625032000
6	10.031340000	5.949511000	3.582232000
1	10.348475000	5.326985000	4.425140000
1	9.213332000	6.600639000	3.917783000
1	10.872742000	6.600666000	3.318541000
6	5.716139000	1.575661000	0.628435000

1	6.089189000	2.335919000	-0.064827000
1	4.861111000	1.075729000	0.157477000
1	6.509531000	0.835208000	0.771215000
6	4.205316000	3.248303000	1.716215000
1	3.835552000	3.683902000	2.653058000
1	3.344401000	2.791261000	1.215028000
1	4.563532000	4.064788000	1.081866000
6	4.747807000	1.098433000	2.879819000
1	5.481509000	0.300439000	3.032751000
1	3.857921000	0.648546000	2.426466000
1	4.461802000	1.486071000	3.863864000
6	5.465249000	5.204558000	7.422135000
1	5.140330000	6.184500000	7.745114000
6	6.795020000	4.708928000	7.527489000
6	6.774118000	3.381392000	7.016537000
1	7.610891000	2.692623000	6.987436000
6	5.448002000	3.073409000	6.613829000
1	5.114786000	2.108398000	6.248539000
6	4.618993000	4.201286000	6.882222000
6	7.963128000	5.374844000	8.245878000
6	3.096147000	4.228766000	6.812152000
6	8.203035000	4.625319000	9.572561000
1	9.023629000	5.087229000	10.134580000
1	8.460586000	3.576496000	9.394222000
1	7.305244000	4.646911000	10.198365000
6	9.243258000	5.305739000	7.398134000
1	9.138087000	5.891865000	6.476824000
1	9.495366000	4.275783000	7.125116000
1	10.096403000	5.721996000	7.946126000
6	7.660278000	6.846370000	8.558895000
1	6.800184000	6.944670000	9.228926000
1	7.449156000	7.415200000	7.647225000
1	8.519736000	7.309748000	9.055618000
6	2.549586000	3.205482000	7.828872000
1	2.894576000	3.441336000	8.840632000
1	2.881541000	2.189863000	7.591016000
1	1.453055000	3.212692000	7.830596000
6	2.594213000	3.851433000	5.409248000
1	1.499198000	3.807999000	5.385910000
1	2.967484000	2.871009000	5.095329000
1	2.903934000	4.601087000	4.670296000
6	2.549032000	5.616210000	7.170622000
1	1.455543000	5.618074000	7.103893000
1	2.931096000	6.383135000	6.490077000
1	2.818293000	5.900713000	8.192937000
92	6.391226000	4.861724000	4.675907000

H. References

1 A. C. Brown, A. B. Altman, T. D. Lohrey, S. Hohloch and J. Arnold, *Chem. Sci.*, 2017, **8**, 5153–5160.
2 W. W. Lukens, S. M. Beshouri, L. L. Blosch, A. L. Stuart and R. A. Andersen, *Organometallics*, 1999, **18**, 1235–1246.
3 M. J. Monreal, R. K. Thomson, T. Cantat, N. E. Travia, B. L. Scott and J. L. Kiplinger, *Organometallics*, 2011, **30**, 2031–2038.
4 T. Cantat, B. L. Scott and J. L. Kiplinger, *Chem. Commun.*, 2010, **46**, 919–921.
5 M. A. Araya, F. A. Cotton, J. H. Matonic and C. A. Murillo, *Inorg. Chem.*, 1995, **34**, 5424–5428.
6 W. Ren, G. Zi, D.-C. Fang and M. D. Walter, *Chem. Eur. J.*, 2011, **17**, 12669–12682.
7 A. Zalkin, A. L. Stuart and R. A. Andersen, *Acta Crystallogr C Cryst Struct Commun*, 1988, **44**, 2106–2108.
8 W. W. Lukens, S. M. Beshouri, A. L. Stuart and R. A. Andersen, *Organometallics*, 1999, **18**, 1247–1252.
9 F. Schulz, V. Sumerin, M. Leskelä, T. Repo and B. Rieger, *Dalton Trans.*, 2010, **39**, 1920–1922.
10 A. J. Rosenthal, M. Devillard, K. Miqueu, G. Bouhadir and D. Bourissou, *Angew. Chem. Int. Ed.*, 2015, **54**, 9198–9202.
11 G. M. Sheldrick, *SADABS, Version 2.03; Bruker Analytical X-Ray Systems*, Inc.: Madison, 2000.
12 S. SAINT, *Area-Detector Integration Program, V. 6.40. Bruker Analytical X-ray Systems*, Inc.: Madison, 2003.
13 G. M. Sheldrick, *Acta Crystallogr C Struct Chem*, 2015, **71**, 3–8.
14 S. Stoll and A. Schweiger, *Journal of Magnetic Resonance*, 2006.
15 A. D. Becke, *J. Chem. Phys.*, 1993, **98**, 5648.
16 J. P. Perdew and Y. Wang, *Phys. Rev. B*, 1992, **45**, 13244–13249.
17 W. Küchle, M. Dolg, H. Stoll and H. Preuss, *J. Chem. Phys.*, 1994, **100**, 7535–7542.
18 X. Cao, M. Dolg and H. Stoll, *J. Chem. Phys.*, 2003, **118**, 487–496.
19 R. Ditchfield, W. J. Hehre and J. A. Pople, *J. Chem. Phys.*, 1971, **54**, 724–728.
20 W. J. Hehre, R. Ditchfield and J. A. Pople, *J. Chem. Phys.*, 1972, **56**, 2257–2261.
21 P. C. Hariharan and J. A. Pople, *Theoret. Chim. Acta*, 1973, **28**, 213–222.
22 R. A. Gaussian 09, M. J. Frisch, G. W. Trucks, H. B. Schlegel, G. E. Scuseria, M. A. Robb, J. R. Cheeseman, G. Scalmani, V. Barone, B. Mennucci, G. A. Petersson, H. Nakatsuji, M. Caricato, X. Li, H. P. Hratchian, A. F. Izmaylov, J. Bloino, G. Zheng, J. L. Sonnenberg, M. Hada, M. Ehara, K. Toyota, R. Fukuda, J. Hasegawa, M. Ishida, T. Nakajima, Y. Honda, O. Kitao, H. Nakai, T. Vreven, J. A. Montgomery, Jr., J. E. Peralta, F. Ogliaro, M. Bearpark, J. J. Heyd, E. Brothers, K. N. Kudin, V. N. Staroverov, R. Kobayashi, J. Normand, K. Raghavachari, A. Rendell, J. C. Burant, S. S. Iyengar, J. Tomasi, M. Cossi, N. Rega, J. M. Millam, M. Klene, J. E. Knox, J. B. Cross, V. Bakken, C. Adamo, J. Jaramillo, R. Gomperts, R. E. Stratmann, O. Yazyev, A. J. Austin, R. Cammi, C. Pomelli, J. W. Ochterski, R. L. Martin, K. Morokuma, V. G. Zakrzewski, G. A. Voth, P. Salvador, J. J. Dannenberg, S. Dapprich, A. D. Daniels, O. Farkas, J. B. Foresman, J. V. Ortiz, J. Cioslowski, D. J. Fox, Gaussian, Inc., Wallingford CT, 2009.

23 J. P. Foster, F. Weinhold, *J. Am. Chem. Soc.*, 1980, **102**, 7211-7218.

24 F. Weinhold, J.E. Carpenter, *The Natural Bond Orbital Lewis Structure Concept for Molecules, Radicals and Radical Ions.* In: R. Naaman, Z. Vager (eds.) *The Structure of Small Molecules and Ions*. Springer: Boston, MA; 1988.

25 R. F. W. Bader *An Introduction to the Electronic Structure of Atoms and Molecules.* Clarke: Toronto, Canada; 1970.

26 T. Lu, F. Chen, *J. Comp. Chem.* 2012, **33**, 580-592.

27 G. A. Zhurko, ChemCraft Home Page: a set of graphical tools for facilitating working with quantum chemistry computations (http://www.chemcraftprog.com).
